# Effects of high doses of vitamin D_3_ on mucosa-associated gut microbiome vary between regions of the human gastrointestinal tract

**DOI:** 10.1007/s00394-015-0966-2

**Published:** 2015-07-01

**Authors:** Mina Bashir, Barbara Prietl, Martin Tauschmann, Selma I. Mautner, Patrizia K. Kump, Gerlies Treiber, Philipp Wurm, Gregor Gorkiewicz, Christoph Högenauer, Thomas R. Pieber

**Affiliations:** Division of Endocrinology and Metabolism, Department of Internal Medicine, Medical University of Graz, Auenbruggerplatz 15, 8036 Graz, Austria; Division of Gastroenterology and Hepatology, Department of Internal Medicine, Medical University of Graz, Graz, Austria; Institute of Pathology, Medical University of Graz, Graz, Austria

**Keywords:** Vitamin D, Human gut microbiome, IBD, Inflammation, Gammaproteobacteria

## Abstract

**Purpose:**

Vitamin D is well known for its effects on bone mineralisation but has also been attributed immunomodulatory properties. It positively influences human health, but in vivo data describing vitamin D effects on the human gut microbiome are missing. We aimed to investigate the effects of oral vitamin D_3_ supplementation on the human mucosa-associated and stool microbiome as well as CD8^+^ T cells in healthy volunteers.

**Methods:**

This was an interventional, open-label, pilot study. Sixteen healthy volunteers (7 females, 9 males) were endoscopically examined to access a total of 7 sites. We sampled stomach, small bowel, colon, and stools before and after 8 weeks of vitamin D_3_ supplementation. Bacterial composition was assessed by pyrosequencing the 16S rRNA gene (V1–2), and CD8^+^ T cell counts were determined by flow cytometry.

**Results:**

Vitamin D_3_ supplementation changed the gut microbiome in the upper GI tract (gastric corpus, antrum, and duodenum). We found a decreased relative abundance of Gammaproteobacteria including *Pseudomonas* spp. and *Escherichia*/*Shigella* spp. and increased bacterial richness. No major changes occurred in the terminal ileum, appendiceal orifice, ascending colon, and sigmoid colon or in stools, but the CD8^+^ T cell fraction was significantly increased in the terminal ileum.

**Conclusion:**

Vitamin D_3_ modulates the gut microbiome of the upper GI tract which might explain its positive influence on gastrointestinal diseases, such as inflammatory bowel disease or bacterial infections. The local effects of vitamin D demonstrate pronounced regional differences in the response of the GI microbiome to external factors, which should be considered in future studies investigating the human microbiome.

**Electronic supplementary material:**

The online version of this article (doi:10.1007/s00394-015-0966-2) contains supplementary material, which is available to authorized users.

## Introduction

The human gut is home to about 10^14^ microorganisms, which are collectively referred to as the gut microbiome. These microorganisms work as biochemical factories, helping the host in nutrient acquisition, vitamin production, and toxin degradation. Bacterial enzymes are also involved in an array of metabolic pathways [1] and can even affect the central nervous system by converting amino acids, carbohydrates, and other ingested substances into bioactive compounds [2]. When it comes to energy acquisition, especially carbohydrate metabolism, the human genome is quite limited and a healthy gut microbiome is necessary to effectively digest the diverse carbohydrates [3].

The human gut microbiome is known to have a major impact on colonisation resistance against intestinal pathogens [4], on modulation of the intestinal immune system [5], and on host metabolism [6, 7]. At the current stage of knowledge, some factors, which influence the composition of the intestinal microbiome, have been identified, including diet [8–10], host genetics [11], environmental circumstances [12], and drugs, such as antibiotics or laxatives [13, 14]. Impairment of gut homoeostasis has been linked to many gastrointestinal diseases, such as inflammatory bowel diseases (IBD), intestinal infections, irritable bowel syndrome (IBS), and colon cancer [15–18] but also to extra-intestinal diseases, such as obesity, diabetes mellitus, autoimmune diseases, and neurological disorders [19]. For general health benefits but also for a number of diseases, supplementation with vitamin D has shown to be beneficial. Vitamin D is well known for its role on bone mineralisation but has also major effects on the immune and the cardiovascular system as well as on host defence against pathogenic microorganisms [15, 20–22]. In many parts of the world, about half of the population is assumed to have insufficient vitamin D levels (lower than 20 ng/mL serum) due to a lack of UV exposure and a diet-lacking vitamin D [23]. Sufficient levels of vitamin D have been associated with a lower risk of autoimmune diseases such as IBD, type 1 diabetes mellitus, and rheumatic diseases, but also neoplasia and infections such as tuberculosis and hepatitis C [21]. Elevating serum vitamin D levels to 42 ng/mL has been estimated to decrease disease rates in various cancers, cardiovascular diseases, diabetes mellitus, and infections by 10–50 % and the overall mortality rate by 18 % per year [24].

The hormonal activity of vitamin D has been attributed to the expression of the vitamin D receptor (VDR), which has its highest expression in CD8^+^ T cells compared with other immune cells [25], and to the vitamin D activating enzyme CYP27B1. VDR and CYP27B1 are found in several different cell types including kidneys, muscle, or prostate, but also in cells of the immune system [25, 26], supporting a prominent role of vitamin D in gut homoeostasis and immunity [25]. Given the beneficial effects of vitamin D, many people are using vitamin D supplementation as part of their regular diet, but effects of vitamin D on the human intestinal microbiome have not yet been studied. We hypothesised that some of the beneficial effects attributed to vitamin D might be mediated by the gastrointestinal microbiome. We recruited healthy human volunteers for an interventional study to assess whether vitamin D_3_ (vitD_3_) affects the human microbiome. Most microbiome studies are limited to easily accessible stool samples, but studies have indicated that there can be a pronounced difference between mucosal and stool microbiomes [13, 27]. Therefore, we sampled seven gastrointestinal regions in addition to stools to assess the mucosa-associated microbiome before and after 8 weeks of oral vitD_3_ supplementation.

## Materials and methods

### Study design, population, and medication

This study was designed as an interventional monocentric open pilot study. The inclusion criteria were: healthy volunteers, age between 18 and 40 years, body mass index (BMI) between 20 and 30 kg/m^2^, and non-smoker. The Ethics Committee at the Medical University of Graz, Austria, approved the study. The study was conducted from January 2012 to August 2012 at the Medical University of Graz according to the principles of Good Clinical Practice (ClinicalTrials.gov, NCT01538485) and ethical standards of the Declaration of Helsinki and all later amendments. All volunteers gave their written informed consent after being informed about the aim of the study, study procedure, and possible risks of participating in this study. Sixteen healthy volunteers, 9 men and 7 women, aged 25 ± 4 (mean ± SD) years participated in the study. All volunteers had a normal BMI (23 ± 3 kg/m^2^) with a mean weight of 69 ± 11 kg and height of 172 ± 8 cm.

After baseline assessment, each volunteer received a weekly dose of 980 IU/kg bodyweight of vitamin D_3_ (Oleovit D_3_, Fresenius Kabi, Graz, Austria) for 4 weeks representing a daily dose of 140 IU/kg bodyweight, but maximal 68,600 IU per week in total. For the remaining 4 weeks, each volunteer received a weekly vitD_3_ dose of 490 IU/kg bodyweight (daily dose of 70 IU/kg bodyweight), but maximal 34,300 IU per week in total.

### Sample collection and storage

**Fig. 1:**
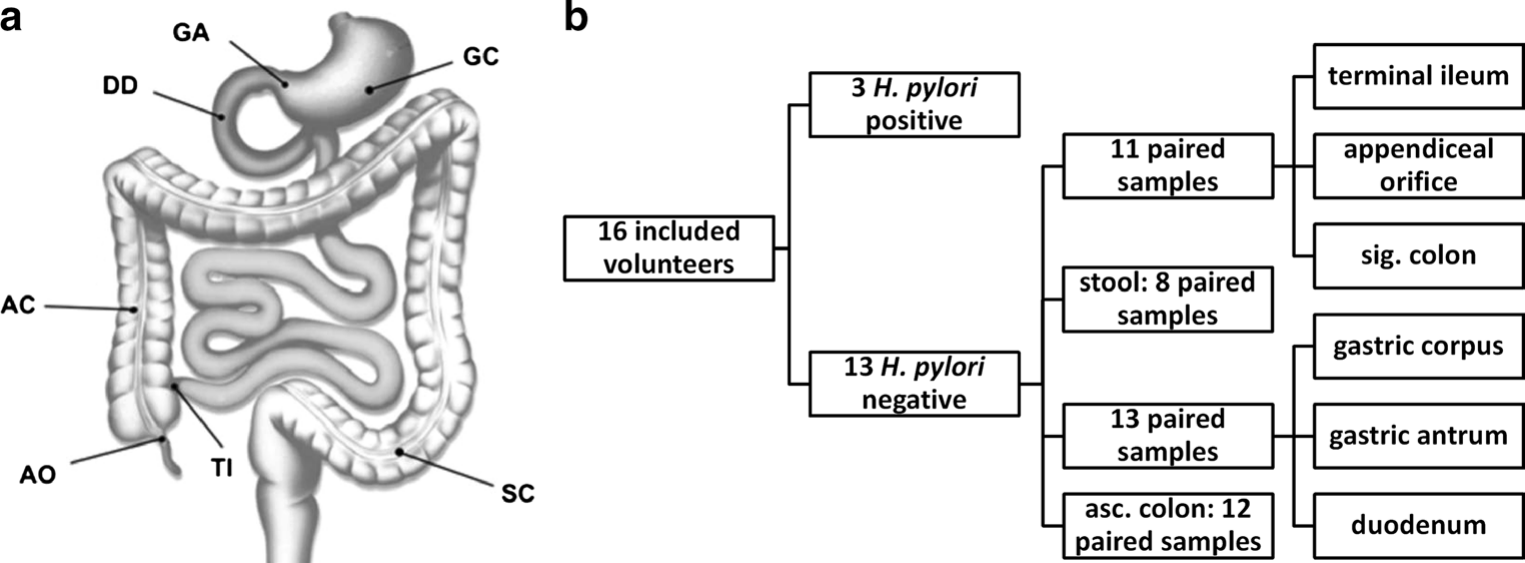
Sampling scheme. **a** Biopsied body regions (drawing generously provided by Tauschmann et al. [46]). **b** Paired samples passed quality filtering. A subgroup of 3 volunteers was analysed separately due to a *H. pylori* infection in their stomach. *GC* gastric corpus, *GA* gastric antrum, *DD* duodenum, *TI* terminal ileum, *AO* appendiceal orifice, *AC* ascending colon, *SC* sigmoid colon

Before vitD_3_ supplementation was started, all volunteers underwent a gastroduodenoscopy followed by a colonoscopy on the next day. Stools were collected before bowel preparation for gastroduodenoscopy and immediately stored at −80 °C until DNA isolation. After 8 weeks of vitD_3_ supplementation, both endoscopic procedures and stool sampling were repeated. As a laxative, all volunteers received 2 litres of MOVIPREP^®^ (Norgine, Marburg, Germany), a polyethylene glycol-based electrolyte solution for bowel preparation. During gastroduodenoscopy, biopsies were taken from the gastric corpus (GC), the gastric antrum (GA), and the descending part of the duodenum (DD) (Fig. 1a). During colonoscopy, biopsies were taken from the terminal ileum (TI), the appendiceal orifice region (AO), the ascending colon (AC) at 10 cm distal to the ileocecal valve, and the sigmoid colon (SC) at 30 cm proximal to the anal canal (Fig. 1a). Two biopsies were taken from each region, immediately frozen and stored at −80 °C until DNA isolation, and two biopsies from each region were immediately placed on ice in complete RPMI media containing 10 % foetal calf serum (cRPMI-10), glutamine, and penicillin/streptomycin (Life Technologies, Darmstadt, Germany) for flow cytometry. Flow cytometry analysis was started within 1 h after the biopsy.

### Isolation of lamina propria mononuclear cells and flow cytometry

Gastrointestinal biopsies were immediately transferred to a dithiothreitol/EDTA solution (Sigma, Hamburg, Germany, and Life Technologies, Darmstadt, Germany) and incubated for 15 min at 37 °C. The samples were finely sliced and digested in a collagenase A solution (Roche, Mannheim, Germany) for 1 h at 37 °C. The resulting cell suspension was passed through a 70-µm cell strainer (BD Biosciences, San Jose, CA, USA) and collected in a cRPMI-10 filled tube (Life Technologies, Darmstadt, Germany). After centrifugation, the pellet was resuspended in PBS (Life Technologies, Darmstadt, Germany) and cell viability was confirmed by staining an aliquot with 0.4 % trypan blue (Sigma, Hamburg, Germany) for microscopy.

For the quantification of CD3^+^CD4^−^CD8^+^ T cells (CD8^+^ T cells) in lamina propria by flow cytometry, the following monoclonal antibodies were used: anti-CD3 PerCP-Cy5.5 (eBiosciences, USA) and anti-CD4 PE and anti-CD8 V500 (BD Biosciences, San Diego, CA, USA). Positive signals were defined by the use of isotype controls and the fluorochrome minus one (FMO) method.

### Vitamin D and safety parameter measurements

The vitamin D levels (calcidiol, 25(OH)D) were measured by using a commercially available enzyme-linked immunosorbent assay (ELISA, IDS, Bolden, UK) with an intra- and inter-assay coefficient of variation of 5.6 and 6.4 %, respectively.

Safety parameters including serum calcium levels, urine calcium, urine calcium/creatinine ratio, complete blood cell count, serum phosphorus, serum albumin, and PTH were assessed at baseline and every 4 weeks thereafter by standard laboratory methods. C-reactive protein was measured by using a Tina quant CRP immunoturbidimetric assay (Roche COBAS INTEGRA, Mannheim, Germany).

### DNA isolation, 16S rRNA gene PCR amplification, and FLX sequencing

Isolation of total genomic DNA, PCR, and FLX sequencing were performed as recently described in Kump et al. [28]. Briefly, a MagnaLyser Instrument (Roche Diagnostics, Mannheim, Germany) and a MagNA Pure LC 2.0 Instrument (Roche Diagnostics) with the MagNA Pure LC DNA Isolation Kit III (bacteria, fungi) were used for automated DNA isolation according to the manufacturer’s instructions. For library preparation, 454 one-way read strategy (Lib-L kit, Primer A, Primer B; Roche 454 Life Science, Branford, CT, USA) amplifying the 16S rRNA hyper variable regions V1–2 with the target specific primers F27-AGAGTTTGATCCTGGCTCAG and R357-CTGCTGCCTYCCGTA was used. FLX fusion primers with FLX adaptor, key, and MID sequences were used for amplification (supplementary table S1). For each sample, a PCR was carried out with 50 ng total genomic DNA in a 25-µL reaction volume, performed in triplicate. Amplicons were purified according to Kump et al. [28] and pooled and sequenced using the GS FLX Titanium Sequencing Kit XLR70 (Roche 454 Life Science, Branford, CT, USA) according to manufacturer’s instructions.

### Sequencing data analysis

Raw sequences were preprocessed and filtered using MOTHUR version 1.31.2 according to the standard Schloss 454 SOP (see http://www.mothur.org/wiki/454_SOP) [29]. In more detail, reads were denoised using PyroNoise [30], chimera filtered with UCHIME [31], pyrosequencing errors were reduced with pre.cluster [32], and non-bacterial sequences were also excluded. High-quality reads were aligned to the SILVA database [33, 34], and taxonomy was assigned by MOTHUR’s implementation of the ribosomal database project (RDP) classifier [35] followed by binning into phylotypes based on taxonomy using a minimum probability cut-off of 80 %. As we were using short reads, we only classified the reads down to the genus level. The shared file, MOTHUR’s OTU-table format was then converted into biom format and passed on to QIIME’s v.1.7 core_diversity.py command using non-phylogenetic parameters [36]. The GC was rarefied to 1178 sequences/sample, GA to 2125, DD to 2441, TI to 1647, AO to 2215, AC to 2014, SC to 2188, and stools to 1788 sequences/sample.

### Statistical analysis

To determine whether phylotypes (e.g. phyla, classes, order) differed significantly between the two time points, we used a paired t test since the study compared samples from the same patients. Subsequently, *P* values were corrected for multivariate testing using the Benjamini–Hochberg false discovery rate (FDR) procedure, as Bonferroni correction is too conservative for sequencing data. Corrected *P* values below 0.05 were considered statistically significant (*P < .05; **P < .01; ***P < .001). To determine whether the intervention had different effects in the biopsied regions, we calculated a Bray–Curtis distance matrix and used an analysis of similarity (ANOSIM) to test for significantly different clustering. ANOSIM was also used to test whether mucosal samples and stools differ significantly. Phylotype richness was compared before and after supplementation by using alpha rarefaction plots based on an observed species matrix and a nonparametric *t* test. The difference between CD8^+^ T cells were tested with a paired *t* test. All values are given as mean ± SD if not otherwise stated.

## Results

Fifteen volunteers completed the whole study. One volunteer could not attend the second colonoscopy due to personal reasons but completed all other study procedures. Vitamin D levels (25(OH)D, calcidiol) increased significantly from 22.3 ± 13.1 ng/mL (before) to 55.2 ± 13.3 ng/mL after vitD_3_ supplementation (*P* < .0001). In all volunteers, all safety parameters, including serum and urine calcium and phosphate, as well as urine calcium/creatinine ratio, remained within a normal range and were not significantly altered (Table 1 and supplementary table S2). Throughout the study, we generated 1003,488 high-quality 16S amplicon reads in the range of 211–380 bp (average 264 bp) from mucosal and stool samples. Five microbiome samples were excluded from sequence data analysis as they were poorly covered (<900 sequences/sample). In a subgroup of 3 volunteers, a gastric *Helicobacter pylori* infection was found during routine clinical processing of biopsies and in the sequencing data. This subgroup was analysed separately as up to 90 % of the observed phylotypes in the GC were classified as *Helicobacter* spp. In total, we analysed 242 well-covered samples, resulting in 13 paired samples (before/after) for the upper GI tract (GC, GA, and DD), 12 paired samples for AC, 11 for TI, AO, and SC, and 8 paired stool samples (Fig. 1b).

**Table 1:** Safety parameters

Safety parameters	Before	After 8 weeks of vitD_3_ supplementation	*P* value
Calcidiol (ng/mL) ± SD	22.3 ± 13.1	55.2 ± 13.3	<0.0001
Calcium (mmol/L) ± SD	2.44 ± 0.09	2.43 ± 0.08	0.75
Ca/creatinine ratio ± SD	0.26 ± 0.18	0.20 ± 0.12	0.24
PTH (pg/mL) ± SD	39.5 ± 12.7	33.5 ± 14.7	0.14

We successfully increased vitamin D levels without significantly altering any of the measured safety parameters. Values are given as mean ± SD (*n* = 16). A paired *t* test was used to calculate *P* values

### Phylogenetic landscape of the human GI tract

**Fig. 2:**
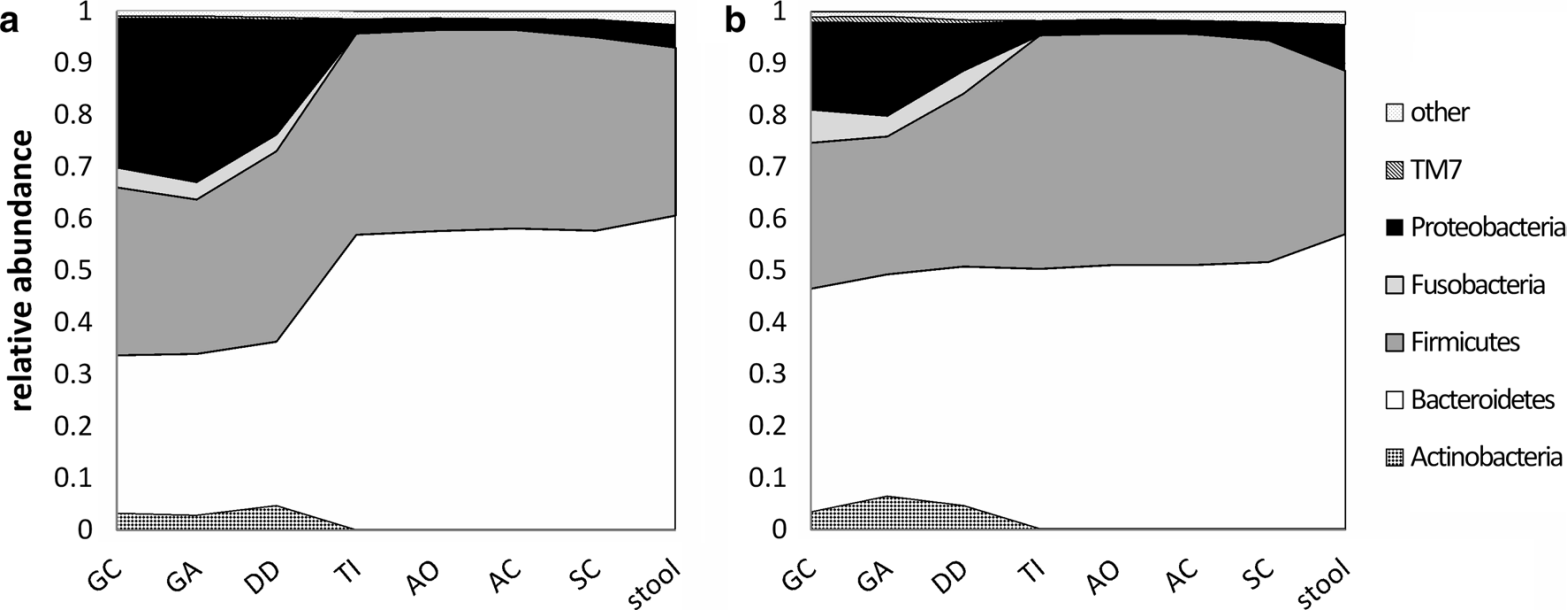
Mean relative abundance of phylogenetic landscape **a** before and **b** after vitD_3_ supplementation in 13 volunteers on the phylum level from the GC to the SC and from stool samples. We see a decline in Proteobacteria and an increase in Bacteroidetes in the upper GI tract (GC, GA, and DD). Lower regions (TI, AO, AC, and SC) and stools did not change on the phylum level. *GC* gastric corpus, *GA* gastric antrum, *DD* duodenum, *TI* terminal ileum, *AO* appendiceal orifice, *AC* ascending colon, *SC* sigmoid colon

Bacteroidetes and Firmicutes were the dominating phyla which we found in the human gut microbiome (Table 2). We found significantly more Proteobacteria, Actinobacteria, TM7, and Fusobacteria in the upper (GC, GA, and DD) compared with the lower GI tract (TI, AO, AC, SC) and stools (*P* < .0001) (supplementary table S3–4). This probably reflects the oral microbial community which is considered the first input to the GI tract. The microbial communities of the TI and the AO region were more similar to the colon rather than the DD. The stool microbiome was more similar to the colon rather than the upper GI tract (Fig. 2a). A list of all found genera in the different regions of the GI tract is provided in supplementary table S5.

**Table 2:** Dominating phyla

	Bacteroidetes	Firmicutes
GC (%)	36.30 ± 7.56	36.93 ± 9.34
GA (%)	37.46 ± 13.44	35.94 ± 12.92
DD (%)	32.06 ± 9.75	38.43 ± 14.48
TI (%)	58.44 ± 8.47	36.52 ± 8.40
AO (%)	57.99 ± 7.23	38.29 ± 8.07
AC (%)	57.86 ± 7.69	38.20 ± 7.57
SC (%)	58.16 ± 7.92	36.50 ± 8.26
Stool (%)	62.87 ± 9.66	31.43 ± 9.90

Values are given as mean ± SD

Stools are often the only samples assessed in microbiome studies. In our study, the mucosal microbiome of the upper GI tract (GC, GA, DD) was significantly different from the stool microbiome (ANOSIM (genus level) *P* < .001). A list of the significantly different genera of the upper GI tract and stools is provided in supplementary table S6. The lower GI microbiome (TI, AO, AC, SC) was not significantly different from stools (ANOSIM (genus level) *P* = 0.105).

To assess the phyla that were affected by vitD_3_ supplementation, we compared the relative phyla abundance before and after 8 weeks of vitD_3_ supplementation (Fig. 2a, b, supplementary table S3). Proteobacteria decreased significantly in the GC (*P* = .0348) and the GA (*P* = .0258), while Bacteroidetes increased significantly in the GC (*P* = .0013) and the DD (*P* = .0013). Other phyla like Actinobacteria, Firmicutes, and Fusobacteria were not significantly affected by vitD_3_ supplementation in any region of the GI tract.

**Fig. 3:**
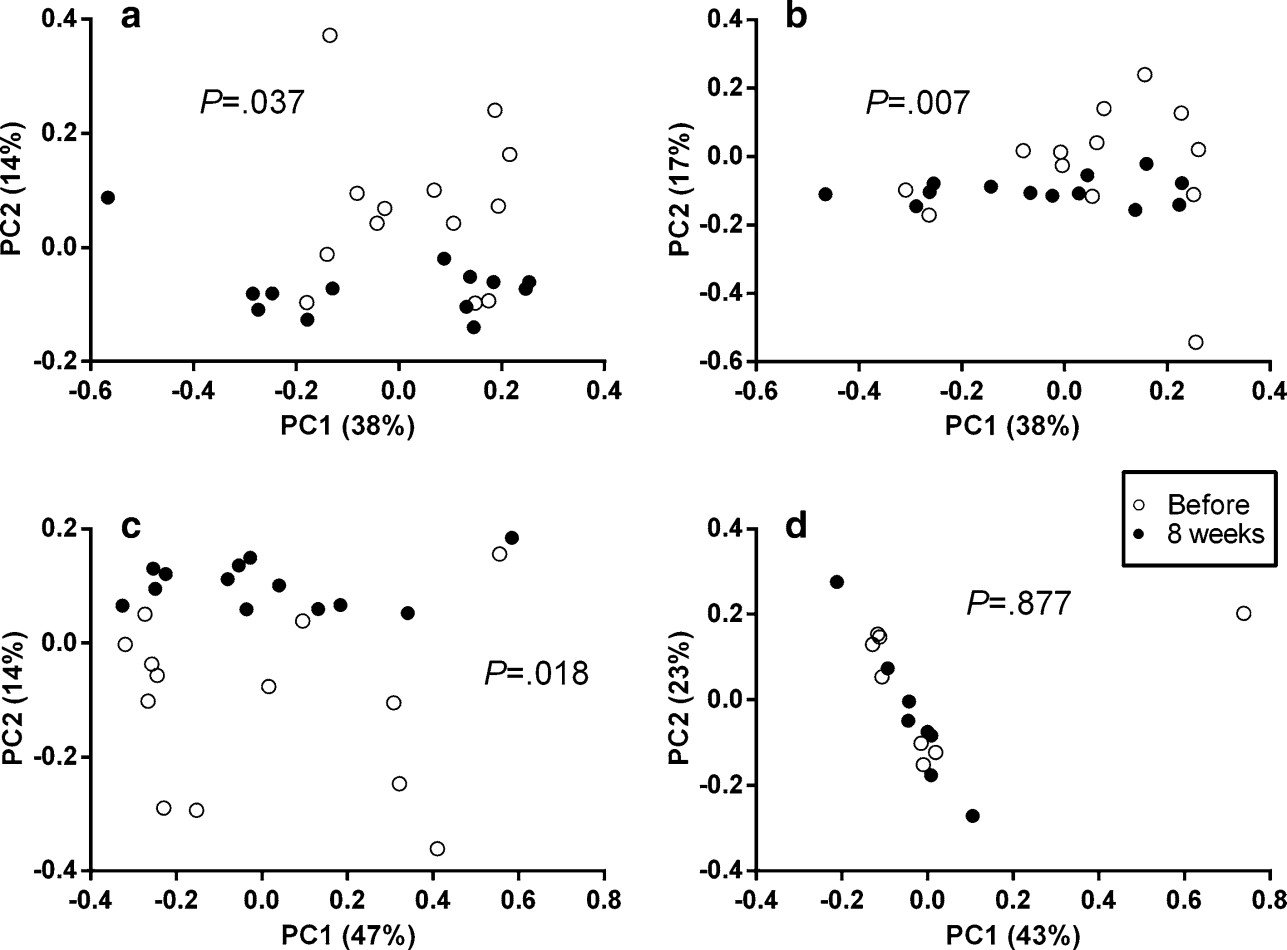
VitD_3_ supplementation changes bacterial community composition and community structure significantly in the upper GI tract in contrast to the lower GI tract. PCoA plots generated on a Bray–Curtis distance matrix on the genus level. **a** GC (*n* = 13), **b** GA (*n* = 13), **c** DD (*n* = 13), **d** stool (*n* = 8). **a**–**c** Distinct separation before and after vitD_3_ supplementation, whereas in stools, all samples cluster together (**d**). ANOSIM was used to test whether community composition is different between the groups. Samples taken before vitD_3_ supplementation are shown in white, and samples taken after supplementation are shown in black. *GC* gastric corpus, *GA* gastric antrum, *DD* duodenum, *TI* terminal ileum, *AO* appendiceal orifice, *AC* ascending colon, *SC* sigmoid colon

Beta-diversity measures represented by PCoA plots show how similar multiple samples are compared to each other. VitD_3_ supplementation changed the community structure in the upper GI tract (GC, GA, and DD samples) indicated by the significant separation of samples before and after vitD_3_ supplementation (Fig. 3a–c). No difference in community structure was observed in stools (Fig. 3d) or mucosal samples from the lower GI tract (TI, AO, AC, SC; see supplementary Fig. S7).

### Reduction in Gammaproteobacteria

**Fig. 4:**
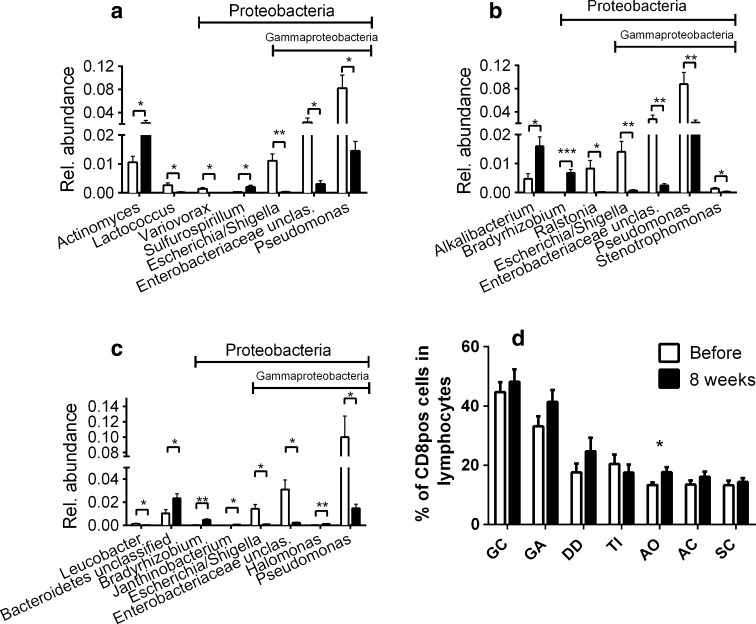
Significantly changed phylotypes (genera) and changes in mucosal CD8^+^ T cell counts after vitD_3_ supplementation. **a** Gastric corpus, **b** gastric antrum, and **c** duodenum. **d** Percentage of CD8^+^ T cells of all lymphocytes isolated from mucosal biopsies (GC, *P* = .5168; GA, *P* = .1317; DD, *P* = .2058; TI, *P* = .5012; AO, *P* = .0315; AC, *P* = .2476; SC, *P* = .5773). Samples taken before vitD_3_ supplementation are shown in *white*, and samples taken after supplementation are shown in *black*. *GC* gastric corpus, *GA* gastric antrum, *DD* duodenum, *TI* terminal ileum, *AO* appendiceal orifice, *AC* ascending colon, *SC* sigmoid colon. *P* < .05 = *; *P* < .01 = **; *P* < .001 = ***; *n* = 13, *bars* = mean ± SEM. A paired *t* test was used to calculate *P* values, which were FDR corrected for multiple testing

To assess whether all Proteobacteria were decreasing or whether just a particular phylotype (i.e. class, order, family or genera) was affected, we monitored the changes on all taxonomic levels in the upper GI tract (GC, GA, DD). Only the class of Gammaproteobacteria (GC, *P* = .045; GA, *P* = .034; DD *P* = .066), including *Pseudomonas* spp. (GC, *P* = .0280; GA, *P* = .0061; DD, *P* = .0316) and *Escherichia*/*Shigella* spp. (GC, *P* = .0022; GA, *P* = .0078; DD, *P* = .0190), showed a significant decline, while other Proteobacteria, like the *Bradyrhizobium* spp. (GA, *P* = .0003; DD, *P* = .0016) and *Sulfurospirillum* spp. (GC, *P* = .033), increased (Fig. 4a–c). Alphaproteobacteria increased significantly in relative abundance after 8 weeks of vitD_3_ supplementation in the GA (Fig. 4b, *P* = .0295). Besides the reduction in Gammaproteobacteria, *Lactococcus* spp. (*P* = .0434) and *Variovorax* spp. (*P* = .0164) decreased significantly. *Actinomyces* spp. (*P* = .0175) increased in the GC, while in the GA *Alkalibacterium* spp. (*P* = .0394) increased and *Ralstonia* spp. (*P* = .0342) decreased in relative abundance. The relative abundance of *Leucobacter* spp. (*P* = .0289) decreased and *Janthinobacterium* spp. (*P* = .0318) increased in the DD. Compared to the upper GI tract, observed changes in the lower GI tract were minor. In the TI, *Roseburia* spp. were significantly increased (*P* = .0421) and *Peptostreptococcus* spp. were decreased (*P* = .0423, supplementary Fig. S8), both representing the phylum Firmicutes. We also found a decline in an unclassified *Clostridia* genus in the AO region (*P* = .0438). Colonic mucosal samples showed no significant changes in any phylotype, whereas in stools, the class of Betaproteobacteria (*P* = .0234) decreased and *Actinomyces* spp. (*P* = .0073) increased in relative abundance (supplementary Fig. S8).

Taken together, our data indicate that vitD_3_ supplementation significantly alters Gammaproteobacteria in the upper GI tract, whereas other GI regions seem to be less affected. CD8^+^ T cells may contribute to the decline in Gammaproteobacteria. Flow cytometry analysis of CD8^+^ T cell counts showed a significant increase in the AO (*P* = .0213) and a trend towards increased CD8^+^ T cells in the other regions after 8 weeks of vitD_3_ supplementation (Fig. 4d).

### VitD_3_ increases richness in the gastric antrum and duodenum

**Fig. 5:**
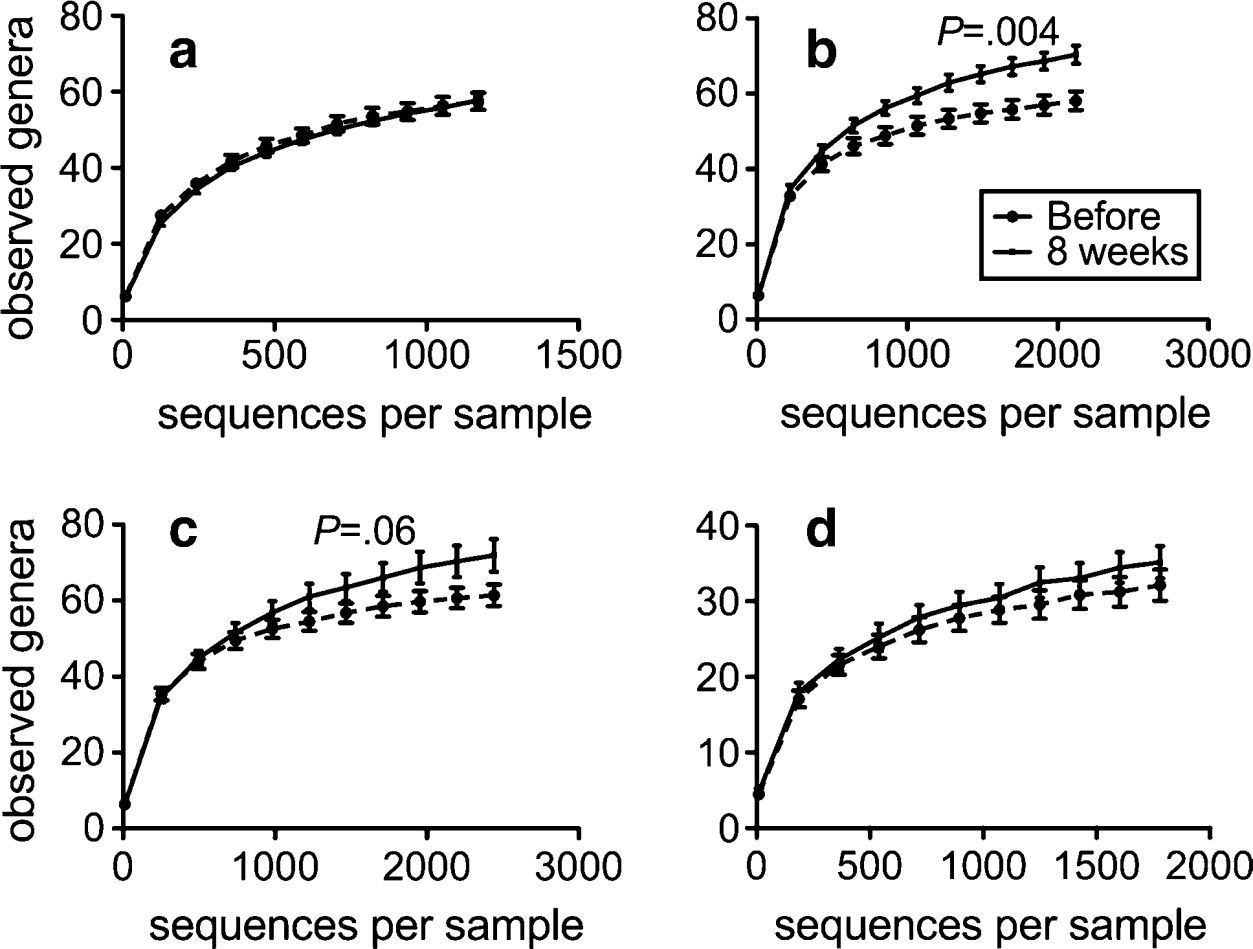
VitD_3_ supplementation increases phylotype richness in the gastric antrum and duodenum; alpha rarefaction curves of **a** the gastric corpus, **b** the gastric antrum, **c** the duodenum, and **d** stool. The number of observed species is plotted against the number of sequences per sample. We see an increase in richness in the gastric antrum (*P* = .004) and a trend in the duodenum (*P* = .06) but not in gastric corpus (*P* = .933) or in stool *(P* = .313). Rarefaction curves also indicate sufficient coverage

Rarefaction analysis indicated sufficient capturing of the microbial diversity in all samples (Fig. 5 and supplementary Fig. S9). After vitD_3_ supplementation, we found a significantly increased phylotype richness in the GA (*P* = .004) and a trend towards an increased phylotype richness in the DD (*P* = .06) (Fig. 5). No change in phylotype richness was seen in GC, TI, AO, AC, SC or in stools (Fig. 5 and supplementary Fig. S9).

### *Helicobacter pylori* reduction after 8 weeks of vitD_3_ supplementation

Microbiome sequence data and routine biopsy-processing indicated *H. pylori* infection in three volunteers. All three volunteers showed an overall decline in *Helicobacter* spp. after 8 weeks of vitD_3_ supplementation. We observed a trend towards a decline in *Helicobacter* spp. in the GA of all 3 volunteers, but changes in the GC and DD were inconsistent (supplementary Fig. S10). When we included the *H. pylori* infected subgroup into the overall microbiome data analysis, we found an additional increase in phylotype richness in the GC after 8 weeks of vitD_3_ supplementation (data not shown).

## Discussion

The results of our study demonstrate the effect of an 8-week high-dose vitD_3_ supplementation on the human microbiome of the upper GI tract. These changes included a reduction in opportunistic pathogens and an increase in bacterial richness.

To the best of our knowledge, the effects of vitD_3_ on the human microbiome have not yet been studied. So far, the effects of a vitamin D supplementation on the gut microbiome have only been studied in murine colitis model. Vitamin D_3_ protected these mice from dextran sodium sulphate-induced colitis, which is a common model of IBD. The severity of colitis was significantly higher in VDR and CYP27B1 knockout mice [37]. Because the composition of the gut microbiome of mice is substantially different from the human microbiome [38], any interventional effects that are found in mouse studies cannot be translated directly to humans.

By taking a series of biopsies from the major regions of the human GI tract, we found an overall increase in phylotype richness together with a decrease in Proteobacteria mostly in the mucosa of the upper GI tract. In particular, the class of Gammaproteobacteria decreased significantly, including genera of typical opportunistic pathogens like *Pseudomonas* spp. and *Escherichia*/*Shigella* spp. Stools and mucosal regions of the lower GI tract showed only minor changes and support recent findings that mucosa-associated microbiomes differ significantly from stools [13] and that only biopsies offer the chance to analyse changes in the mucosa-associated microbiome in sufficient detail. Most human intestinal microbiome studies are based on data obtained from stools [8, 9, 16, 17, 39] or rectal/colonic mucosal samples [13, 18, 40]. These studies have investigated external influences, such as the effects of diet [8, 9, 41, 42], physical exercise [39], or drugs [14], and have identified associations with certain disease like IBD, IBS, or obesity [8, 18, 40]. But some interventional studies using therapeutics which affect the microbiome (e.g. prebiotics, omega-3 fatty acids) were not able to identify significant changes in stools [43, 44] since some effects might be limited to the upper GI tract. With our comprehensive sampling of the whole GI tract, we were able to show that the major effects of vitamin D on the human gastrointestinal microbiome were only present in the upper GI tract but not in mucosal samples of the colon, ileum, or stools. Future microbiome studies need to be aware that interventions such as vitamin D supplementation can have significantly different effects on different regions of the GI tract and that these effects cannot necessarily be detected in stools.

In general, the assessment of the microbial composition of samples from the gastrointestinal system is often affected by high inter-individual variation [45] and the methods used for sample processing (e.g. sample storage, DNA extraction, and primer choice). Previous studies have shown that freezing of stools prior to DNA extraction can lead to an increased ratio of Firmicutes to Bacteroidetes [46]. The proportion of Firmicutes reported in the literature varies considerably. Some studies report a microbial composition dominated by Firmicutes [27, 47, 48], whereas other studies report similar levels compared to the results in our study [45, 49, 50].

Our study also showed that the intestinal microbiome varies along the human GI tract and thus supports a previous study which found a similar bacterial distribution pattern in the ileum and colon of healthy humans [51]. The bacterial composition along the GI tract is determined by different niche factors (e.g. pH, oxygen, tension, nutrients), and microbiome data do not necessarily reflect the anatomical categorisation. In our study, the microbiome of the duodenum was more similar to the stomach rather than to the terminal ileum, which was more similar to colon samples. Different regions of the human GI tract vary in function, permeability, type, and number of immune cells [52, 53].

A significantly higher number of CD8^+^ T cells in the upper GI (GC and GA) compared with lower GI sites [52] is part of the first defence line against foodborne pathogens. The significant decrease in Gammaproteobacteria that we found was limited to the mucosa of the upper GI tract and might have been driven by the increased CD8^+^ T cell fraction found after 8 weeks of vitD_3_ supplementation. CD8^+^ T cells have the highest expression level of VDR compared with other immune cells [25], which suggests that CD8^+^ T cell function is also regulated by vitamin D. High vitamin D levels have been associated with a decline in naïve CD8^+^ T cells and an increase in CD8^+^ effector T cells [54]. After vitD_3_ supplementation, we observed a trend towards elevated CD8^+^ T cell levels in all seven assessed GI regions, but we did not analyse the different CD8^+^ T cell subpopulations in this study. The stimulation of mucosal CD8^+^ T cells by vitD_3_ could contribute for the effects on Gammaproteobacteria together with other known effects of vitamin D, such as the induction of antimicrobial peptides (e.g. cathelicidin [55]) and the stimulation of certain cytokine production by mucosal dendritic cells [56]. In addition, murine CD8^+^ T cells [57] and human activated T cells [58] have been found to express 1α-hydroxylase (CYP27B1) which enables the production of calcitriol, the active hormonal state of vitD_3_. Calcitriol influences cytokine production in human CD8^+^ T cells [59] and is known to act as a stop signal in inflammatory processes [60, 61]. CD8^+^ T cells are also capable of directly destroying harmed or infected host cells, thus lowering the number of proinflammatory cells [62].

Reducing such an inflammatory environment by vitD_3_ could diminish the competitive advantage of opportunistic pathogens, such as *Escherichia*/*Shigella* spp. or *Pseudomonas* spp. which are evolutionary better adapted to inflammation and can outcompete commensal bacteria [63]. In return, a low inflammatory environment allows beneficial bacteria such as Bacteroidetes to outcompete opportunistic pathogens, resulting in increased phylotype richness which we found in this study.

An increase in bacterial richness could be particularly beneficial for gastrointestinal problems such non-alcoholic steatohepatitis or IBD which is characterised by decreased bacterial richness, lower vitamin D levels, and an overgrowth of pathogens [17, 50, 64–68]. Studies have reported beneficial effects of a vitamin D treatment in IBD patients [69] which could be to some extent due to the restoration of gut microbiome homoeostasis indicated by a reduction in opportunistic pathogens such as Enterobacteriaceae, which belong to the class of Gammaproteobacteria [17, 65–67, 70]. 16S rRNA amplicon sequencing as used in our study yields only relative abundances which cannot be used to differentiate between an increase in Bacteroidia and a decrease in Gammaproteobacteria. We assume that a decrease in Gammaproteobacteria is more likely because we found a decrease in several genera of Gammaproteobacteria, whereas no single genus of Bacteroidia was significantly altered after 8 weeks of vitD_3_ supplementation.

After vitD_3_ supplementation, we also found a decrease in overall abundance of *Helicobacter* spp. in the *H. pylori*-positive subgroup where approximately 90 % of all bacteria in the stomach were classified as *Helicobacter* spp. These are the first data to describe an effect of vitD_3_ on *H. pylori* infections and support the finding that CYP27B1 knockout mice which cannot produce calcitriol show a significantly higher relative abundance of Helicobacteriaceae compared to wild-type mice [37]. In these knockout mice, a calcitriol supplementation successfully decreased Helicobacteriaceae levels. The relevant role of vitD_3_ in *H. pylori* infections is also supported by the finding that *H. pylori* itself induces increased expression of the VDR [71].

VitD_3_ dosing in our study was above currently recommended dosing for vitamin D supplementation [72] and resulted in mean vitamin D levels of 55.2 ng/mL. The safety of such a high vitamin D supplementation has previously been reported [73]. We did not observed any adverse effects and no safety parameters, including serum and urine calcium and phosphate were significantly changed during 8 weeks of a high-dose vitD_3_ supplementation. At present, it is not advised to generally administer such high vitD_3_ doses for longer time periods in humans. Randomized intervention long-term trials are needed to evaluate the risks and benefits of such high vitD_3_ doses for human health, because this dose is not what the international guidelines state as recommended daily allowance. One limitation of our study is the relatively small sample size mainly because of the invasive procedures. Despite the relatively small study cohort and a high degree of inter-individual variation of the intestinal microbiome, we were able to observe a significant modulatory effect of vitD_3_ in the upper GI tract.

## Conclusion

The marked reduction in Gammaproteobacteria, which include typical opportunistic pathogens and the increase in phylotype richness, supports the beneficial effect of a high-dose vitD_3_ supplementation on the human gut microbiome. This might in part explain the effects of a vitamin D-rich diet on IBD or bacterial infections and encourage studying the effects of vitamin D in these patients. The fact that the vitamin D effect on the microbiome was only evident in the upper GI tract should guide future microbiome studies in humans to also assess the upper GI tract in addition to stools and colonic mucosal samples.

## Electronic supplementary material

Below is the link to the electronic supplementary material.

Supplementary material 1 (DOCX 17657 kb)
