# Supplementary material for: Effects of high doses of vitamin D3 on mucosa-associated gut microbiome vary between regions of the human gastrointestinal tract
Source: Eur J Nutr. 2015 Jul 1;55:1479–89. doi: 10.1007/s00394-015-0966-2 (PMC4875045; doi:10.1007/s00394-015-0966-2)
Supplement: Supplementary file 1 — Supplementary material 1 (DOCX 17657 kb) [file 394_2015_966_MOESM1_ESM.docx]

S1: Used Multiplex Identifiers (Roche): each sample was tagged with a unique MID (barcode), which was used to demultiplex samples.

| Nr. | **sample** | **Roche-MID** | **Roche-MID sequence** |
| --- | --- | --- | --- |
| 1 | 301 after app. orfice | MID 1 | ACGAGTGCGT |
| 2 | 301 after sig. colon | MID 2 | TGTACTACTC |
| 3 | 301 after asc. colon | MID 3 | ACTGTACAGT |
| 4 | 301 after t. ileum | MID 4 | AGACTATACT |
| 5 | 302 after app. orfice | MID 5 | TCGATCACGT |
| 6 | 302 after sig. colon | MID 6 | TCGATCACGT |
| 7 | 302 after asc. colon | MID 7 | TCGATCACGT |
| 8 | 302 after t. ileum | MID 8 | TCGATCACGT |
| 9 | 303 after app. orfice | MID 10 | ACGAGTGCGT |
| 10 | 303 after sig. colon | MID 11 | ACGAGTGCGT |
| 11 | 303 after asc. colon | MID 13 | ACGAGTGCGT |
| 12 | 303 after t. ileum | MID 14 | ACGAGTGCGT |
| 13 | 304 after app. orfice | MID 15 | ACGAGTGCGT |
| 14 | 304 after sig. colon | MID 16 | TCACGTACTA |
| 15 | 304 after asc. colon | MID 17 | CGTCTAGTAC |
| 16 | 304 after t. ileum | MID 18 | TCTACGTAGC |
| 17 | 305 after app. orfice | MID 19 | TGTACTACTC |
| 18 | 305 after sig. colon | MID 20 | ACGACTACAG |
| 19 | 305 after asc. colon | MID 21 | CGTAGACTAG |
| 20 | 305 after t. ileum | MID 22 | TACGAGTATG |
| 21 | 306 after app. orfice | MID 23 | TACTCTCGTG |
| 22 | 306 after sig. colon | MID 24 | TAGAGACGAG |
| 23 | 306 after asc. colon | MID 25 | TCGTCGCTCG |
| 24 | 306 after t. ileum | MID 26 | ACATACGCGT |
| 25 | 307 after app. orfice | MID 27 | ACGCGAGTAT |
| 26 | 307 after sig. colon | MID 28 | ACTACTATGT |
| 27 | 307 after asc. colon | MID 29 | ACTGTACAGT |
| 28 | 307 after t. ileum | MID 30 | AGACTATACT |
| 29 | 308 after app. orfice | MID 31 | AGACTATACT |
| 30 | 308 after sig. colon | MID 32 | AGACTATACT |
| 31 | 308 after asc. colon | MID 1 | ACGAGTGCGT |
| 32 | 308 after t. ileum | MID 2 | TGTACTACTC |
| 33 | 309 after app. orfice | MID 3 | ACTGTACAGT |
| 34 | 309 after sig. colon | MID 4 | AGACTATACT |
| 35 | 309 after asc. colon | MID 5 | TCGATCACGT |
| 36 | 309 after t. ileum | MID 6 | TCGATCACGT |
| 37 | 313 after app. orfice | MID 7 | TCGATCACGT |
| 38 | 313 after sig. colon | MID 8 | TCGATCACGT |
| 39 | 313 after asc. colon | MID 10 | ACGAGTGCGT |
| 40 | 313 after t. ileum | MID 11 | ACGAGTGCGT |
| 41 | 315 after app. orfice | MID 13 | ACGAGTGCGT |
| 42 | 315 after sig. colon | MID 14 | ACGAGTGCGT |
| 43 | 315 after asc. colon | MID 15 | ACGAGTGCGT |
| 44 | 315 after t. ileum | MID 16 | TCACGTACTA |
| 45 | 316 after app. orfice | MID 17 | CGTCTAGTAC |
| 46 | 316 after sig. colon | MID 18 | TCTACGTAGC |
| 47 | 316 after asc. colon | MID 19 | TGTACTACTC |
| 48 | 316 after t. ileum | MID 20 | ACGACTACAG |
| 49 | 317 after app. orfice | MID 21 | CGTAGACTAG |
| 50 | 317 after sig. colon | MID 22 | TACGAGTATG |
| 51 | 317 after asc. colon | MID 23 | TACTCTCGTG |
| 52 | 317 after t. ileum | MID 24 | TAGAGACGAG |
| 53 | 318 after app. orfice | MID 25 | TCGTCGCTCG |
| 54 | 318 after sig. colon | MID 26 | ACATACGCGT |
| 55 | 318 after asc. colon | MID 27 | ACGCGAGTAT |
| 56 | 318 after t. ileum | MID 28 | ACTACTATGT |
| 57 | 319 after app. orfice | MID 29 | ACTGTACAGT |
| 58 | 319 after sig. colon | MID 30 | AGACTATACT |
| 59 | 319 after asc. colon | MID 31 | AGACTATACT |
| 60 | 319 after t. ileum | MID 32 | AGACTATACT |
| 61 | 301 after gastric corpus | MID 1 | ACGAGTGCGT |
| 62 | 301 after gastric antrum | MID 2 | TGTACTACTC |
| 63 | 301 after duodenum | MID 3 | ACTGTACAGT |
| 64 | 302 after gastric corpus | MID 4 | AGACTATACT |
| 65 | 302 after gastric antrum | MID 5 | TCGATCACGT |
| 66 | 302 after duodenum | MID 6 | TCGATCACGT |
| 67 | 303 after gastric corpus | MID 7 | TCGATCACGT |
| 68 | 303 after gastric antrum | MID 8 | TCGATCACGT |
| 69 | 303 after duodenum | MID 10 | ACGAGTGCGT |
| 70 | 304 after gastric corpus | MID 11 | ACGAGTGCGT |
| 71 | 304 after gastric antrum | MID 13 | ACGAGTGCGT |
| 72 | 304 after duodenum | MID 14 | ACGAGTGCGT |
| 73 | 305 after gastric corpus | MID 15 | ACGAGTGCGT |
| 74 | 305 after gastric antrum | MID 16 | TCACGTACTA |
| 75 | 305 after duodenum | MID 17 | CGTCTAGTAC |
| 76 | 306 after gastric corpus | MID 18 | TCTACGTAGC |
| 77 | 306 after gastric antrum | MID 19 | TGTACTACTC |
| 78 | 306 after duodenum | MID 20 | ACGACTACAG |
| 79 | 307 after gastric corpus | MID 21 | CGTAGACTAG |
| 80 | 307 after gastric antrum | MID 22 | TACGAGTATG |
| 81 | 307 after duodenum | MID 23 | TACTCTCGTG |
| 82 | 308 after gastric corpus | MID 24 | TAGAGACGAG |
| 83 | 308 after gastric antrum | MID 25 | TCGTCGCTCG |
| 84 | 308 after duodenum | MID 26 | ACATACGCGT |
| 85 | 309 after gastric corpus | MID 27 | ACGCGAGTAT |
| 86 | 309 after gastric antrum | MID 28 | ACTACTATGT |
| 87 | 309 after duodenum | MID 29 | ACTGTACAGT |
| 88 | 310 after gastric corpus | MID 30 | AGACTATACT |
| 89 | 310 after gastric antrum | MID 31 | AGACTATACT |
| 90 | 310 after duodenum | MID 32 | AGACTATACT |
| 91 | 313 after gastric corpus | MID 1 | ACGAGTGCGT |
| 92 | 313 after gastric antrum | MID 2 | TGTACTACTC |
| 93 | 313 after duodenum | MID 3 | ACTGTACAGT |
| 94 | 315 V gastric corpus | MID 4 | AGACTATACT |
| 95 | 315 after gastric antrum | MID 5 | TCGATCACGT |
| 96 | 315 after duodenum | MID 6 | TCGATCACGT |
| 97 | 316 after gastric corpus | MID 7 | TCGATCACGT |
| 98 | 316 after gastric antrum | MID 8 | TCGATCACGT |
| 99 | 316 after duodenum | MID 10 | ACGAGTGCGT |
| 100 | 317 after gastric corpus | MID 11 | ACGAGTGCGT |
| 101 | 317 after gastric antrum | MID 13 | ACGAGTGCGT |
| 102 | 317 after duodenum | MID 14 | ACGAGTGCGT |
| 103 | 318 after gastric corpus | MID 15 | ACGAGTGCGT |
| 104 | 318 after gastric antrum | MID 16 | TCACGTACTA |
| 105 | 318 after duodenum | MID 17 | CGTCTAGTAC |
| 106 | 319 after gastric corpus | MID 18 | TCTACGTAGC |
| 107 | 319 after gastric antrum | MID 19 | TGTACTACTC |
| 108 | 319 after duodenum | MID 20 | ACGACTACAG |
| 109 | 301 after stool | MID 21 | CGTAGACTAG |
| 110 | 302 after stool | MID 22 | TACGAGTATG |
| 111 | 303 after stool | MID 23 | TACTCTCGTG |
| 112 | 304 after stool | MID 24 | TAGAGACGAG |
| 113 | 306 after stool | MID 25 | TCGTCGCTCG |
| 114 | 313 after stool | MID 26 | ACATACGCGT |
| 115 | 315 after stool | MID 27 | ACGCGAGTAT |
| 116 | 316 after stool | MID 28 | ACTACTATGT |
| 117 | 317 after stool | MID 29 | ACTGTACAGT |
| 118 | 318 after stool | MID 30 | AGACTATACT |
| 119 | 319 after stool | MID 31 | AGACTATACT |
| 120 | 301 before gastric antrum | MID 1 | ACGAGTGCGT |
| 121 | 301 before gastric corpus | MID 2 | ACGCTCGACA |
| 122 | 301 before duodenum | MID 3 | AGACGCACTC |
| 123 | 301 before t. ileum | MID 4 | AGCACTGTAG |
| 124 | 301 before app. orfice | MID 5 | ATCAGACACG |
| 125 | 301 before asc. colon | MID 6 | ATATCGCGAG |
| 126 | 301 before sig. colon | MID 7 | CGTGTCTCTA |
| 127 | 302 before gastric antrum | MID 8 | CTCGCGTGTC |
| 128 | 302 before gastric corpus | MID 31 | AGCGTCGTCT |
| 129 | 302 before duodenum | MID 10 | TCTCTATGCG |
| 130 | 302 before t. ileum | MID 11 | TGATACGTCT |
| 131 | 302 before app. orfice | MID 32 | AGTACGCTAT |
| 132 | 302 before sig. colon | MID 13 | CATAGTAGTG |
| 133 | 302 before asc. colon | MID 14 | CGAGAGATAC |
| 134 | 303 before gastric antrum | MID 15 | ATACGACGTA |
| 135 | 303 before gastric corpus | MID 16 | TCACGTACTA |
| 136 | 303 before duodenum | MID 17 | CGTCTAGTAC |
| 137 | 303 before t. ileum | MID 18 | TCTACGTAGC |
| 138 | 303 before app. orfice | MID 19 | TGTACTACTC |
| 139 | 303 before asc. colon | MID 20 | ACGACTACAG |
| 140 | 303 before sig. colon | MID 21 | CGTAGACTAG |
| 141 | 304 before gastric corpus | MID 22 | TACGAGTATG |
| 142 | 304 before gastric antrum | MID 23 | TACTCTCGTG |
| 143 | 304 before duodenum | MID 24 | TAGAGACGAG |
| 144 | 304 before t. ileum | MID 25 | TCGTCGCTCG |
| 145 | 304 before app. orfice | MID 26 | ACATACGCGT |
| 146 | 304 before asc. colon | MID 27 | ACGCGAGTAT |
| 147 | 304 before sig. colon | MID 28 | ACTACTATGT |
| 148 | 305 before gastric antrum | MID 29 | ACTGTACAGT |
| 149 | 305 before gastric corpus | MID 30 | AGACTATACT |
| 150 | 305 before duodenum | MID 41 | TAGTGTAGAT |
| 151 | 305 before t. ileum | MID 42 | TCGATCACGT |
| 152 | 305 before app. orfice | MID 1 | ACGAGTGCGT |
| 153 | 305 before asc. colon | MID 2 | ACGCTCGACA |
| 154 | 305 before sig. colon | MID 3 | AGACGCACTC |
| 155 | 306 before gastric antrum | MID 4 | AGCACTGTAG |
| 156 | 306 before gastric corpus | MID 5 | ATCAGACACG |
| 157 | 306 before duodenum | MID 6 | ATATCGCGAG |
| 158 | 306 before t. ileum | MID 7 | CGTGTCTCTA |
| 159 | 306 before app. orfice | MID 8 | CTCGCGTGTC |
| 160 | 306 before asc. colon | MID 31 | AGCGTCGTCT |
| 161 | 306 before sig. colon | MID 10 | TCTCTATGCG |
| 162 | 307 before gastric antrum | MID 11 | TGATACGTCT |
| 163 | 307 before gastric corpus | MID 32 | AGTACGCTAT |
| 164 | 307 before duodenum | MID 13 | CATAGTAGTG |
| 165 | 307 before t. ileum | MID 14 | CGAGAGATAC |
| 166 | 307 before app. orfice | MID 15 | ATACGACGTA |
| 167 | 307 before asc. colon | MID 16 | TCACGTACTA |
| 168 | 307 before sig. colon | MID 17 | CGTCTAGTAC |
| 169 | 308 before gastric antrum | MID 18 | TCTACGTAGC |
| 170 | 308 before gastric corpus | MID 19 | TGTACTACTC |
| 171 | 308 before duodenum | MID 20 | ACGACTACAG |
| 172 | 308 before t. ileum | MID 21 | CGTAGACTAG |
| 173 | 308 before app. orfice | MID 22 | TACGAGTATG |
| 174 | 308 before asc. colon | MID 23 | TACTCTCGTG |
| 175 | 308 before sig. colon | MID 24 | TAGAGACGAG |
| 176 | 309 before gastric antrum | MID 25 | TCGTCGCTCG |
| 177 | 309 before gastric corpus | MID 26 | ACATACGCGT |
| 178 | 309 before duodenum | MID 27 | ACGCGAGTAT |
| 179 | 309 before t. ileum | MID 28 | ACTACTATGT |
| 180 | 309 before app. orfice | MID 29 | ACTGTACAGT |
| 181 | 309 before asc. colon | MID 30 | AGACTATACT |
| 182 | 309 before sig. colon | MID 41 | TAGTGTAGAT |
| 183 | 310 before gastric antrum | MID 42 | TCGATCACGT |
| 184 | 310 before gastric corpus | MID 1 | ACGAGTGCGT |
| 185 | 310 before duodenum | MID 2 | ACGCTCGACA |
| 186 | 310 before t. ileum | MID 3 | AGACGCACTC |
| 187 | 310 before app. orfice | MID 4 | AGCACTGTAG |
| 188 | 310 before asc. colon | MID 5 | ATCAGACACG |
| 189 | 310 before sig. colon | MID 6 | ATATCGCGAG |
| 190 | 313 before gastric antrum | MID 7 | CGTGTCTCTA |
| 191 | 313 before gastric corpus | MID 8 | CTCGCGTGTC |
| 192 | 313 before duodenum | MID 31 | AGCGTCGTCT |
| 193 | 313 before t. ileum | MID 10 | TCTCTATGCG |
| 194 | 313 before app. orfice | MID 11 | TGATACGTCT |
| 195 | 313 before asc. colon | MID 32 | AGTACGCTAT |
| 196 | 313 before sig. colon | MID 13 | CATAGTAGTG |
| 197 | 315 before gastric antrum | MID 14 | CGAGAGATAC |
| 198 | 315 before gastric corpus | MID 15 | ATACGACGTA |
| 199 | 315 before duodenum | MID 16 | TCACGTACTA |
| 200 | 315 before t. ileum | MID 17 | CGTCTAGTAC |
| 201 | 315 before app. orfice | MID 18 | TCTACGTAGC |
| 202 | 315 before asc. colon | MID 19 | TGTACTACTC |
| 203 | 315 before sig. colon | MID 20 | ACGACTACAG |
| 204 | 316 before gastric antrum | MID 21 | CGTAGACTAG |
| 205 | 316 before gastric corpus | MID 22 | TACGAGTATG |
| 206 | 316 before duodenum | MID 23 | TACTCTCGTG |
| 207 | 316 before t. ileum | MID 24 | TAGAGACGAG |
| 208 | 316 before app. orfice | MID 25 | TCGTCGCTCG |
| 209 | 316 before asc. colon | MID 26 | ACATACGCGT |
| 210 | 316 before sig. colon | MID 27 | ACGCGAGTAT |
| 211 | 317 before gastric antrum | MID 28 | ACTACTATGT |
| 212 | 317 before gastric corpus | MID 29 | ACTGTACAGT |
| 213 | 317 before duodenum | MID 30 | AGACTATACT |
| 214 | 317 before t. ileum | MID 41 | TAGTGTAGAT |
| 215 | 317 before app. orfice | MID 42 | TCGATCACGT |
| 216 | 317 before asc. colon | MID 1 | ACGAGTGCGT |
| 217 | 317 before sig. colon | MID 2 | ACGCTCGACA |
| 218 | 318 before gastric antrum | MID 3 | AGACGCACTC |
| 219 | 318 before gastric corpus | MID 4 | AGCACTGTAG |
| 220 | 318 before duodenum | MID 5 | ATCAGACACG |
| 221 | 318 before t. ileum | MID 6 | ATATCGCGAG |
| 222 | 318 before app. orfice | MID 7 | CGTGTCTCTA |
| 223 | 318 before asc. colon | MID 8 | CTCGCGTGTC |
| 224 | 318 before sig. colon | MID 31 | AGCGTCGTCT |
| 225 | 319 before gastric antrum | MID 10 | TCTCTATGCG |
| 226 | 319 before gastric corpus | MID 11 | TGATACGTCT |
| 227 | 319 before duodenum | MID 32 | AGTACGCTAT |
| 228 | 319 before t. ileum | MID 13 | CATAGTAGTG |
| 229 | 319 before app. orfice | MID 14 | CGAGAGATAC |
| 230 | 319 before asc. colon | MID 15 | ATACGACGTA |
| 231 | 319 before sig. colon | MID 16 | TCACGTACTA |
| 232 | 301 before stool | MID 17 | CGTCTAGTAC |
| 233 | 302 before stool | MID 18 | TCTACGTAGC |
| 234 | 303 before stool | MID 19 | TGTACTACTC |
| 235 | 304 before stool | MID 20 | ACGACTACAG |
| 236 | 305 before stool | MID 21 | CGTAGACTAG |
| 237 | 306 before stool | MID 22 | TACGAGTATG |
| 238 | 307 before stool | MID 23 | TACTCTCGTG |
| 239 | 308 before stool | MID 24 | TAGAGACGAG |
| 240 | 309 before stool | MID 25 | TCGTCGCTCG |
| 241 | 310 before stool | MID 26 | ACATACGCGT |
| 242 | 313 before stool | MID 27 | ACGCGAGTAT |
| 243 | 315 before stool | MID 28 | ACTACTATGT |
| 244 | 316 before stool | MID 29 | ACTGTACAGT |
| 245 | 317 before stool | MID 30 | AGACTATACT |
| 246 | 318 before stool | MID 41 | TAGTGTAGAT |
| 247 | 319 before stool | MID 42 | TCGATCACGT |

**S2: Safety parameter**

|  | **before** | | | | **safety visit** | | | | **after 8 weeks of vitD_3_ supplementation** | | | |
| --- | --- | --- | --- | --- | --- | --- | --- | --- | --- | --- | --- | --- |
| volunteer | Calcidiol [ng/mL] | Calcium [mmol/L] | Ca/Creatinin ratio | PTH [pg/mL] | Calcidiol [ng/mL] | Calcium [mmol/L] | Ca/Creatinin ratio | PTH [pg/mL] | Calcidiol [ng/mL] | Calcium [mmol/L] | Ca/Creatinin ratio | PTH [pg/mL] |
| **301** | 16.1 | 2.4 | 0.14 | 33.6 | 76.1 | 2.23 | 0.06 | 34.9 | 51.4 | 2.25 | 0.2 | 45 |
| **302** | 17.8 | 2.5 | 0.4 | 62.4 | 64.1 | 2.41 | 0.23 | 43.5 | 48 | 2.47 | 0.29 | 75.8 |
| **303** | 8.6 | 2.36 | 0.3 | 38.1 | 67.6 | 2.35 | 0.11 | 47.5 | 54.1 | 2.37 | 0.06 | 32.9 |
| **304** | 18.1 | 2.3 | 0.21 | 41.8 | 73 | 2.45 | 0.1 | 22.4 | 66.6 | 2.51 | 0.04 | 23 |
| **305** | 16.4 | 2.57 | 0.54 | 21.3 | 44.3 | 2.43 | 0.35 | 29.9 | 33.1 | 2.5 | 0.24 | 34.7 |
| **306** | 7.2 | 2.58 | 0.06 | 35.9 | 64.3 | 2.42 | 0.06 | 34.6 | 55.5 | 2.43 | 0.11 | 34.5 |
| **307** | 46.1 | 2.41 | 0.73 | 24 | 74.3 | 2.39 | 0.19 | 30.8 | 65.5 | 2.38 | 0.26 | 20.4 |
| **308** | 42.4 | 2.48 | 0.14 | 45.8 | 94.5 | 2.33 | 0.12 | 11.8 | 59.1 | 2.37 | 0.2 | 27.8 |
| **309** | 24.5 | 2.43 | 0.18 | 38.1 | 53.7 | 2.6 | 0.15 | 25.3 | 43.9 | 2.46 | 0.49 | 21.8 |
| **310** | 19.6 | 2.28 | 0.31 | 43.2 | 58.9 | 2.36 | 0.34 | 59 | 42.2 | 2.35 | 0.05 | 40.3 |
| **313** | 11.2 | 2.38 | 0.31 | 48.6 | 77.3 | 2.51 | 0.07 | 42.6 | 71.9 | 2.35 | 0.08 | 19 |
| **315** | 8.9 | 2.41 | 0.13 | 36.2 | 41.8 | 2.47 | 0.22 | 30.1 | 38.7 | 2.54 | 0.21 | 28.9 |
| **316** | 44.7 | 2.39 | 0.06 | 27.9 | 85.6 | - | - | 28.9 | 73.8 | 2.39 | 0.26 | 24.1 |
| **317** | 20.7 | 2.43 | 0.25 | 64 | 64.9 | 2.34 | 0.29 | 39.5 | 50.1 | 2.41 | 0.12 | 23.2 |
| **318** | 37.1 | 2.55 | 0.09 | 48.7 | 70.1 | 2.45 | 0.1 | 68.4 | 79.3 | 2.55 | 0.22 | 53.3 |
| **319** | 16.7 | 2.5 | 0.3 | 22.8 | 66.3 | 2.48 | 0.2 | 23.8 | 50.2 | 2.52 | 0.31 | 30.8 |
| **mean** | **22.3** | **2.4** | **0.26** | **39.5** | **67.3** | **2.4** | **0.17** | **35.8** | **55.2** | **2.4** | **0.20** | **33.5** |
| ***P*-value** |  |  |  |  |  |  |  |  | **<0.0001** | **0.7531** | **0.2447** | **0.1381** |

S3: Mean relative abundances of phyla before and after 8 weeks of vitD_3_ supplementation in the different regions of the GI-tract.

*Significantly affected phyla (*P*<.05).

All other phyla were not significantly affected.

**S4: Comparing lower and upper GI’s mean relative abundances of phyla**

| Taxon | upper GI* | lower GI^+^ | FDR cor. *P*-value |
| --- | --- | --- | --- |
| Actinobacteria | 0.040398764 | 0.001199235 | 5.49E-23 |
| Bacteroidetes | 0.351007792 | 0.583232274 | 1.72E-20 |
| Fusobacteria | 0.038526838 | 0.000673727 | 1.05E-18 |
| Proteobacteria | 0.182012102 | 0.026746975 | 1.15E-15 |
| TM7 | 0.005354578 | 8.08E-05 | 4.65E-13 |
| Firmicutes | 0.372404336 | 0.368892662 | 0.86930121 |
| unclassified | 0.007422402 | 0.019079958 | 0.00203004 |

*Upper GI tract (GC; GA, DD)

^+^Lower GI tract (TI, AO; AC; SC, stool)

**S5: Mean relative abundance of genera in investigated gastrointestinal regions**

| **Taxon** | **GC** | **GA** | **DD** | **TI** | **AO** | **AC** | **SC** | **stool** |
| --- | --- | --- | --- | --- | --- | --- | --- | --- |
| **Acidobacteria;Acidobacteria Gp16** | 0.00% | 0.03% | 0.00% | 0.00% | 0.00% | 0.00% | 0.00% | 0.00% |
| **Acidobacteria;Acidobacteria Gp2** | 0.02% | 0.04% | 0.00% | 0.00% | 0.00% | 0.00% | 0.01% | 0.00% |
| **Actinobacteria;Actinobacteria;Actinomycetales;Actinomycetaceae;Actinomyces** | 0.93% | 1.44% | 1.41% | 0.04% | 0.00% | 0.02% | 0.01% | 0.01% |
| **Actinobacteria;Actinobacteria;Actinomycetales;Actinomycetaceae;Mobiluncus** | 0.00% | 0.00% | 0.03% | 0.00% | 0.00% | 0.00% | 0.00% | 0.00% |
| **Actinobacteria;Actinobacteria;Actinomycetales;Actinomycetaceae;unclassified** | 0.01% | 0.00% | 0.01% | 0.00% | 0.00% | 0.00% | 0.00% | 0.00% |
| **Actinobacteria;Actinobacteria;Actinomycetales;Cellulomonadaceae;Tropheryma** | 0.03% | 0.00% | 0.03% | 0.00% | 0.00% | 0.00% | 0.00% | 0.00% |
| **Actinobacteria;Actinobacteria;Actinomycetales;Corynebacteriaceae;Corynebacterium** | 0.04% | 0.02% | 0.12% | 0.00% | 0.00% | 0.01% | 0.00% | 0.00% |
| **Actinobacteria;Actinobacteria;Actinomycetales;Corynebacteriaceae;unclassified** | 0.01% | 0.00% | 0.00% | 0.00% | 0.00% | 0.00% | 0.00% | 0.00% |
| **Actinobacteria;Actinobacteria;Actinomycetales;Dermabacteraceae;Brachybacterium** | 0.00% | 0.07% | 0.00% | 0.00% | 0.00% | 0.00% | 0.00% | 0.00% |
| **Actinobacteria;Actinobacteria;Actinomycetales;Dermacoccaceae;Dermacoccus** | 0.00% | 0.00% | 0.01% | 0.00% | 0.00% | 0.00% | 0.00% | 0.00% |
| **Actinobacteria;Actinobacteria;Actinomycetales;Dietziaceae;Dietzia** | 0.01% | 0.00% | 0.00% | 0.00% | 0.00% | 0.00% | 0.00% | 0.00% |
| **Actinobacteria;Actinobacteria;Actinomycetales;Intrasporangiaceae;Janibacter** | 0.01% | 0.00% | 0.00% | 0.00% | 0.00% | 0.00% | 0.00% | 0.00% |
| **Actinobacteria;Actinobacteria;Actinomycetales;Microbacteriaceae;Amnibacterium** | 0.01% | 0.00% | 0.00% | 0.00% | 0.00% | 0.00% | 0.00% | 0.00% |
| **Actinobacteria;Actinobacteria;Actinomycetales;Microbacteriaceae;Curtobacterium** | 0.01% | 0.01% | 0.00% | 0.00% | 0.00% | 0.00% | 0.00% | 0.00% |
| **Actinobacteria;Actinobacteria;Actinomycetales;Microbacteriaceae;Leifsonia** | 0.00% | 0.01% | 0.00% | 0.00% | 0.00% | 0.00% | 0.00% | 0.00% |
| **Actinobacteria;Actinobacteria;Actinomycetales;Microbacteriaceae;Leucobacter** | 0.14% | 0.15% | 0.13% | 0.00% | 0.00% | 0.00% | 0.01% | 0.00% |
| **Actinobacteria;Actinobacteria;Actinomycetales;Microbacteriaceae;Microbacterium** | 0.01% | 0.01% | 0.01% | 0.00% | 0.00% | 0.00% | 0.00% | 0.00% |
| **Actinobacteria;Actinobacteria;Actinomycetales;Microbacteriaceae;Zimmermannella** | 0.01% | 0.00% | 0.00% | 0.00% | 0.00% | 0.00% | 0.00% | 0.00% |
| **Actinobacteria;Actinobacteria;Actinomycetales;Microbacteriaceae;unclassified** | 0.07% | 0.01% | 0.09% | 0.00% | 0.00% | 0.00% | 0.00% | 0.00% |
| **Actinobacteria;Actinobacteria;Actinomycetales;Micrococcaceae;Kocuria** | 0.01% | 0.01% | 0.00% | 0.00% | 0.00% | 0.00% | 0.00% | 0.00% |
| **Actinobacteria;Actinobacteria;Actinomycetales;Micrococcaceae;Micrococcus** | 0.07% | 0.00% | 0.03% | 0.00% | 0.00% | 0.01% | 0.00% | 0.00% |
| **Actinobacteria;Actinobacteria;Actinomycetales;Micrococcaceae;Rothia** | 0.43% | 0.30% | 0.67% | 0.01% | 0.00% | 0.00% | 0.00% | 0.00% |
| **Actinobacteria;Actinobacteria;Actinomycetales;Micrococcaceae;unclassified** | 0.00% | 0.01% | 0.00% | 0.00% | 0.00% | 0.00% | 0.00% | 0.00% |
| **Actinobacteria;Actinobacteria;Actinomycetales;Mycobacteriaceae;Mycobacterium** | 0.01% | 0.00% | 0.03% | 0.00% | 0.00% | 0.00% | 0.00% | 0.00% |
| **Actinobacteria;Actinobacteria;Actinomycetales;Nakamurellaceae;Humicoccus** | 0.01% | 0.00% | 0.00% | 0.00% | 0.00% | 0.00% | 0.00% | 0.00% |
| **Actinobacteria;Actinobacteria;Actinomycetales;Nocardiaceae;Rhodococcus** | 0.18% | 0.12% | 0.37% | 0.00% | 0.00% | 0.00% | 0.00% | 0.00% |
| **Actinobacteria;Actinobacteria;Actinomycetales;Nocardioidaceae;Nocardioides** | 0.02% | 0.01% | 0.00% | 0.00% | 0.00% | 0.00% | 0.00% | 0.00% |
| **Actinobacteria;Actinobacteria;Actinomycetales;Nocardioidaceae;unclassified** | 0.01% | 0.00% | 0.00% | 0.00% | 0.00% | 0.00% | 0.00% | 0.00% |
| **Actinobacteria;Actinobacteria;Actinomycetales;Propionibacteriaceae;Microlunatus** | 0.03% | 0.00% | 0.00% | 0.00% | 0.00% | 0.00% | 0.00% | 0.00% |
| **Actinobacteria;Actinobacteria;Actinomycetales;Propionibacteriaceae;Propionibacterium** | 1.06% | 0.74% | 1.27% | 0.01% | 0.03% | 0.01% | 0.02% | 0.00% |
| **Actinobacteria;Actinobacteria;Actinomycetales;Propionibacteriaceae;unclassified** | 0.00% | 0.00% | 0.01% | 0.00% | 0.00% | 0.00% | 0.00% | 0.00% |
| **Actinobacteria;Actinobacteria;Actinomycetales;Pseudonocardiaceae;Pseudonocardia** | 0.01% | 0.00% | 0.00% | 0.00% | 0.00% | 0.00% | 0.00% | 0.00% |
| **Actinobacteria;Actinobacteria;Actinomycetales;Streptomycetaceae;Streptomyces** | 0.03% | 0.00% | 0.01% | 0.00% | 0.01% | 0.00% | 0.00% | 0.00% |
| **Actinobacteria;Actinobacteria;Actinomycetales;unclassified;unclassified** | 0.02% | 0.05% | 0.00% | 0.00% | 0.00% | 0.00% | 0.00% | 0.00% |
| **Actinobacteria;Actinobacteria;Bifidobacteriales;Bifidobacteriaceae;Bifidobacterium** | 0.03% | 0.00% | 0.00% | 0.01% | 0.00% | 0.00% | 0.00% | 0.01% |
| **Actinobacteria;Actinobacteria;Coriobacteriales;Coriobacteriaceae;Asaccharobacter** | 0.00% | 0.00% | 0.00% | 0.01% | 0.00% | 0.01% | 0.01% | 0.01% |
| **Actinobacteria;Actinobacteria;Coriobacteriales;Coriobacteriaceae;Atopobium** | 0.65% | 0.49% | 0.53% | 0.06% | 0.00% | 0.01% | 0.00% | 0.01% |
| **Actinobacteria;Actinobacteria;Coriobacteriales;Coriobacteriaceae;Collinsella** | 0.00% | 0.03% | 0.00% | 0.06% | 0.04% | 0.04% | 0.06% | 0.01% |
| **Actinobacteria;Actinobacteria;Coriobacteriales;Coriobacteriaceae;Coriobacterium** | 0.00% | 0.00% | 0.00% | 0.01% | 0.00% | 0.00% | 0.00% | 0.00% |
| **Actinobacteria;Actinobacteria;Coriobacteriales;Coriobacteriaceae;Eggerthella** | 0.00% | 0.00% | 0.01% | 0.00% | 0.00% | 0.00% | 0.00% | 0.02% |
| **Actinobacteria;Actinobacteria;Coriobacteriales;Coriobacteriaceae;Olsenella** | 0.00% | 0.01% | 0.00% | 0.01% | 0.01% | 0.01% | 0.00% | 0.00% |
| **Actinobacteria;Actinobacteria;Coriobacteriales;Coriobacteriaceae;unclassified** | 0.00% | 0.00% | 0.00% | 0.00% | 0.01% | 0.01% | 0.00% | 0.00% |
| **Actinobacteria;Actinobacteria;unclassified;unclassified;unclassified** | 0.00% | 0.00% | 0.03% | 0.00% | 0.00% | 0.00% | 0.00% | 0.00% |
| **Bacteroidetes;Ohtaekwangia** | 0.01% | 0.00% | 0.00% | 0.00% | 0.00% | 0.00% | 0.00% | 0.00% |
| **Bacteroidetes;Bacteroidia;Bacteroidales;Bacteroidaceae;Bacteroides** | 11.47% | 12.39% | 15.42% | 46.63% | 47.19% | 46.89% | 45.81% | 47.09% |
| **Bacteroidetes;Bacteroidia;Bacteroidales;Porphyromonadaceae;Barnesiella** | 0.07% | 0.01% | 0.07% | 0.45% | 0.73% | 0.56% | 0.65% | 0.63% |
| **Bacteroidetes;Bacteroidia;Bacteroidales;Porphyromonadaceae;Butyricimonas** | 0.00% | 0.00% | 0.01% | 0.10% | 0.16% | 0.05% | 0.10% | 0.06% |
| **Bacteroidetes;Bacteroidia;Bacteroidales;Porphyromonadaceae;Odoribacter** | 0.06% | 0.01% | 0.01% | 0.26% | 0.23% | 0.15% | 0.35% | 0.17% |
| **Bacteroidetes;Bacteroidia;Bacteroidales;Porphyromonadaceae;Paludibacter** | 0.00% | 0.03% | 0.04% | 0.00% | 0.00% | 0.00% | 0.00% | 0.00% |
| **Bacteroidetes;Bacteroidia;Bacteroidales;Porphyromonadaceae;Parabacteroides** | 0.16% | 0.35% | 0.18% | 1.54% | 1.95% | 1.51% | 1.61% | 1.85% |
| **Bacteroidetes;Bacteroidia;Bacteroidales;Porphyromonadaceae;Porphyromonas** | 1.14% | 0.75% | 0.72% | 0.00% | 0.01% | 0.00% | 0.02% | 0.00% |
| **Bacteroidetes;Bacteroidia;Bacteroidales;Porphyromonadaceae;Tannerella** | 0.04% | 0.05% | 0.03% | 0.00% | 0.00% | 0.00% | 0.00% | 0.00% |
| **Bacteroidetes;Bacteroidia;Bacteroidales;Porphyromonadaceae;unclassified** | 0.00% | 0.00% | 0.05% | 0.13% | 0.11% | 0.10% | 0.05% | 0.05% |
| **Bacteroidetes;Bacteroidia;Bacteroidales;Prevotellaceae;Hallella** | 0.00% | 0.00% | 0.01% | 0.00% | 0.00% | 0.00% | 0.00% | 0.00% |
| **Bacteroidetes;Bacteroidia;Bacteroidales;Prevotellaceae;Paraprevotella** | 0.00% | 0.01% | 0.01% | 0.06% | 0.08% | 0.12% | 0.14% | 0.17% |
| **Bacteroidetes;Bacteroidia;Bacteroidales;Prevotellaceae;Prevotella** | 19.60% | 19.45% | 12.96% | 4.34% | 3.75% | 4.58% | 5.55% | 9.62% |
| **Bacteroidetes;Bacteroidia;Bacteroidales;Prevotellaceae;unclassified** | 0.15% | 0.35% | 0.07% | 0.20% | 0.13% | 0.23% | 0.24% | 0.12% |
| **Bacteroidetes;Bacteroidia;Bacteroidales;Rikenellaceae;Alistipes** | 0.00% | 0.06% | 0.16% | 0.53% | 0.59% | 0.54% | 0.76% | 1.13% |
| **Bacteroidetes;Bacteroidia;Bacteroidales;unclassified;unclassified** | 0.50% | 0.87% | 0.56% | 0.94% | 1.01% | 0.83% | 1.06% | 0.24% |
| **Bacteroidetes;Flavobacteria;Flavobacteriales;Flavobacteriaceae;Capnocytophaga** | 0.10% | 0.08% | 0.04% | 0.00% | 0.00% | 0.00% | 0.00% | 0.00% |
| **Bacteroidetes;Flavobacteria;Flavobacteriales;Flavobacteriaceae;Chryseobacterium** | 0.07% | 0.18% | 0.21% | 0.00% | 0.00% | 0.01% | 0.00% | 0.00% |
| **Bacteroidetes;Flavobacteria;Flavobacteriales;Flavobacteriaceae;Cloacibacterium** | 0.24% | 0.12% | 0.05% | 0.00% | 0.00% | 0.00% | 0.00% | 0.00% |
| **Bacteroidetes;Flavobacteria;Flavobacteriales;Flavobacteriaceae;Elizabethkingia** | 0.00% | 0.00% | 0.01% | 0.00% | 0.00% | 0.00% | 0.00% | 0.00% |
| **Bacteroidetes;Flavobacteria;Flavobacteriales;Flavobacteriaceae;Flavobacterium** | 0.06% | 0.04% | 0.18% | 0.00% | 0.00% | 0.00% | 0.00% | 0.00% |
| **Bacteroidetes;Flavobacteria;Flavobacteriales;Flavobacteriaceae;unclassified** | 0.18% | 0.16% | 0.10% | 0.00% | 0.00% | 0.00% | 0.00% | 0.00% |
| **Bacteroidetes;Sphingobacteria;Sphingobacteriales;Chitinophagaceae;unclassified** | 0.01% | 0.00% | 0.03% | 0.00% | 0.00% | 0.00% | 0.00% | 0.00% |
| **Bacteroidetes;Sphingobacteria;Sphingobacteriales;Cytophagaceae;Dyadobacter** | 0.01% | 0.00% | 0.00% | 0.00% | 0.00% | 0.00% | 0.00% | 0.00% |
| **Bacteroidetes;Sphingobacteria;Sphingobacteriales;Cytophagaceae;Hymenobacter** | 0.01% | 0.00% | 0.01% | 0.00% | 0.00% | 0.00% | 0.00% | 0.00% |
| **Bacteroidetes;Sphingobacteria;Sphingobacteriales;Cytophagaceae;unclassified** | 0.04% | 0.00% | 0.01% | 0.00% | 0.00% | 0.00% | 0.00% | 0.00% |
| **Bacteroidetes;Sphingobacteria;Sphingobacteriales;Sphingobacteriaceae;Pedobacter** | 0.02% | 0.01% | 0.01% | 0.00% | 0.00% | 0.00% | 0.00% | 0.00% |
| **Bacteroidetes;Sphingobacteria;Sphingobacteriales;Sphingobacteriaceae;Sphingobacterium** | 0.08% | 0.03% | 0.01% | 0.00% | 0.00% | 0.00% | 0.00% | 0.00% |
| **Bacteroidetes;unclassified;unclassified;unclassified;unclassified** | 1.55% | 2.74% | 1.08% | 1.90% | 1.97% | 1.83% | 1.12% | 0.49% |
| **Deinococcus-Thermus;Deinococci;Deinococcales;Deinococcaceae;Deinococcus** | 0.00% | 0.01% | 0.02% | 0.00% | 0.01% | 0.00% | 0.00% | 0.00% |
| **Deinococcus-Thermus;Deinococci;Thermales;Thermaceae;Thermus** | 0.00% | 0.03% | 0.00% | 0.00% | 0.00% | 0.00% | 0.00% | 0.00% |
| **Firmicutes;Bacilli;Bacillales;Bacillaceae_1;Anoxybacillus** | 0.01% | 0.00% | 0.01% | 0.00% | 0.00% | 0.01% | 0.00% | 0.00% |
| **Firmicutes;Bacilli;Bacillales;Bacillaceae_1;Bacillus** | 0.05% | 0.03% | 0.03% | 0.00% | 0.00% | 0.00% | 0.00% | 0.00% |
| **Firmicutes;Bacilli;Bacillales;Bacillaceae_1;Geobacillus** | 0.00% | 0.04% | 0.00% | 0.00% | 0.00% | 0.00% | 0.00% | 0.00% |
| **Firmicutes;Bacilli;Bacillales;Bacillales_Incertae_Sedis_XI;Gemella** | 1.41% | 1.51% | 1.93% | 0.14% | 0.02% | 0.01% | 0.01% | 0.00% |
| **Firmicutes;Bacilli;Bacillales;Paenibacillaceae_1;Paenibacillus** | 0.03% | 0.01% | 0.00% | 0.00% | 0.00% | 0.00% | 0.00% | 0.00% |
| **Firmicutes;Bacilli;Bacillales;Planococcaceae;Planococcaceae_incertae_sedis** | 0.00% | 0.01% | 0.00% | 0.00% | 0.00% | 0.00% | 0.00% | 0.00% |
| **Firmicutes;Bacilli;Bacillales;Staphylococcaceae;Macrococcus** | 0.00% | 0.00% | 0.05% | 0.00% | 0.00% | 0.00% | 0.00% | 0.00% |
| **Firmicutes;Bacilli;Bacillales;Staphylococcaceae;Staphylococcus** | 0.20% | 0.14% | 0.45% | 0.00% | 0.00% | 0.01% | 0.00% | 0.00% |
| **Firmicutes;Bacilli;Bacillales;Thermoactinomycetaceae_1;Thermoactinomyces** | 0.00% | 0.00% | 0.04% | 0.00% | 0.00% | 0.00% | 0.00% | 0.00% |
| **Firmicutes;Bacilli;Lactobacillales;Aerococcaceae;Abiotrophia** | 0.02% | 0.03% | 0.08% | 0.00% | 0.00% | 0.00% | 0.01% | 0.00% |
| **Firmicutes;Bacilli;Lactobacillales;Carnobacteriaceae;Alkalibacterium** | 0.77% | 0.59% | 0.80% | 0.00% | 0.00% | 0.00% | 0.00% | 0.00% |
| **Firmicutes;Bacilli;Lactobacillales;Carnobacteriaceae;Carnobacterium** | 0.01% | 0.04% | 0.40% | 0.00% | 0.00% | 0.00% | 0.00% | 0.00% |
| **Firmicutes;Bacilli;Lactobacillales;Carnobacteriaceae;Dolosigranulum** | 0.01% | 0.00% | 0.00% | 0.00% | 0.00% | 0.00% | 0.00% | 0.00% |
| **Firmicutes;Bacilli;Lactobacillales;Carnobacteriaceae;Granulicatella** | 1.76% | 1.51% | 2.00% | 0.10% | 0.04% | 0.01% | 0.02% | 0.00% |
| **Firmicutes;Bacilli;Lactobacillales;Carnobacteriaceae;unclassified** | 0.03% | 0.00% | 0.00% | 0.00% | 0.00% | 0.00% | 0.00% | 0.00% |
| **Firmicutes;Bacilli;Lactobacillales;Enterococcaceae;Enterococcus** | 0.04% | 0.03% | 0.03% | 0.00% | 0.00% | 0.00% | 0.00% | 0.01% |
| **Firmicutes;Bacilli;Lactobacillales;Enterococcaceae;Vagococcus** | 0.00% | 0.07% | 0.01% | 0.00% | 0.00% | 0.00% | 0.00% | 0.00% |
| **Firmicutes;Bacilli;Lactobacillales;Lactobacillaceae;Lactobacillus** | 0.13% | 0.11% | 0.17% | 0.00% | 0.00% | 0.00% | 0.02% | 0.00% |
| **Firmicutes;Bacilli;Lactobacillales;Lactobacillaceae;Pediococcus** | 0.00% | 0.01% | 0.00% | 0.00% | 0.00% | 0.00% | 0.00% | 0.00% |
| **Firmicutes;Bacilli;Lactobacillales;Streptococcaceae;Lactococcus** | 0.30% | 0.13% | 0.35% | 0.00% | 0.00% | 0.00% | 0.00% | 0.01% |
| **Firmicutes;Bacilli;Lactobacillales;Streptococcaceae;Streptococcus** | 18.79% | 15.20% | 17.77% | 0.79% | 0.17% | 0.14% | 0.16% | 0.29% |
| **Firmicutes;Clostridia;Clostridiales;Clostridiaceae_1;Clostridium_sensu_stricto** | 0.06% | 0.05% | 0.05% | 0.00% | 0.01% | 0.00% | 0.03% | 0.27% |
| **Firmicutes;Clostridia;Clostridiales;Clostridiaceae_1;unclassified** | 0.00% | 0.00% | 0.01% | 0.00% | 0.00% | 0.00% | 0.00% | 0.03% |
| **Firmicutes;Clostridia;Clostridiales;Clostridiales_Incertae_Sedis_XI;Finegoldia** | 0.01% | 0.00% | 0.00% | 0.00% | 0.00% | 0.00% | 0.00% | 0.00% |
| **Firmicutes;Clostridia;Clostridiales;Clostridiales_Incertae_Sedis_XI;Parvimonas** | 0.00% | 0.04% | 0.05% | 0.00% | 0.00% | 0.00% | 0.01% | 0.00% |
| **Firmicutes;Clostridia;Clostridiales;Clostridiales_Incertae_Sedis_XI;Peptoniphilus** | 0.05% | 0.02% | 0.02% | 0.00% | 0.00% | 0.00% | 0.00% | 0.00% |
| **Firmicutes;Clostridia;Clostridiales;Clostridiales_Incertae_Sedis_XIII;Anaerovorax** | 0.00% | 0.00% | 0.00% | 0.01% | 0.01% | 0.01% | 0.01% | 0.00% |
| **Firmicutes;Clostridia;Clostridiales;Clostridiales_Incertae_Sedis_XIII;Mogibacterium** | 0.14% | 0.05% | 0.10% | 0.01% | 0.00% | 0.00% | 0.00% | 0.00% |
| **Firmicutes;Clostridia;Clostridiales;Clostridiales_Incertae_Sedis_XIII;unclassified** | 0.00% | 0.01% | 0.00% | 0.00% | 0.01% | 0.01% | 0.00% | 0.01% |
| **Firmicutes;Clostridia;Clostridiales;Eubacteriaceae;Anaerofustis** | 0.00% | 0.00% | 0.00% | 0.00% | 0.00% | 0.01% | 0.00% | 0.00% |
| **Firmicutes;Clostridia;Clostridiales;Eubacteriaceae;Eubacterium** | 0.46% | 0.50% | 0.66% | 0.01% | 0.01% | 0.00% | 0.00% | 0.00% |
| **Firmicutes;Clostridia;Clostridiales;Eubacteriaceae;unclassified** | 0.08% | 0.05% | 0.04% | 0.00% | 0.00% | 0.00% | 0.00% | 0.00% |
| **Firmicutes;Clostridia;Clostridiales;Lachnospiraceae;Anaerostipes** | 0.00% | 0.00% | 0.00% | 0.00% | 0.00% | 0.00% | 0.01% | 0.05% |
| **Firmicutes;Clostridia;Clostridiales;Lachnospiraceae;Blautia** | 1.19% | 0.50% | 0.34% | 3.98% | 3.69% | 4.05% | 4.21% | 1.10% |
| **Firmicutes;Clostridia;Clostridiales;Lachnospiraceae;Butyrivibrio** | 0.00% | 0.00% | 0.00% | 0.00% | 0.00% | 0.00% | 0.00% | 0.01% |
| **Firmicutes;Clostridia;Clostridiales;Lachnospiraceae;Catonella** | 0.05% | 0.06% | 0.04% | 0.00% | 0.00% | 0.00% | 0.00% | 0.00% |
| **Firmicutes;Clostridia;Clostridiales;Lachnospiraceae;Clostridium_XlVa** | 0.08% | 0.04% | 0.09% | 0.69% | 0.53% | 0.48% | 0.64% | 0.25% |
| **Firmicutes;Clostridia;Clostridiales;Lachnospiraceae;Clostridium_XlVb** | 0.00% | 0.03% | 0.00% | 0.04% | 0.01% | 0.05% | 0.04% | 0.05% |
| **Firmicutes;Clostridia;Clostridiales;Lachnospiraceae;Coprococcus** | 0.05% | 0.26% | 0.08% | 1.07% | 1.06% | 1.06% | 0.98% | 0.29% |
| **Firmicutes;Clostridia;Clostridiales;Lachnospiraceae;Dorea** | 0.06% | 0.16% | 0.04% | 0.19% | 0.14% | 0.18% | 0.18% | 0.14% |
| **Firmicutes;Clostridia;Clostridiales;Lachnospiraceae;Lachnospiracea_incertae_sedis** | 0.62% | 0.86% | 1.06% | 7.58% | 6.95% | 8.83% | 7.33% | 5.50% |
| **Firmicutes;Clostridia;Clostridiales;Lachnospiraceae;Moryella** | 0.18% | 0.12% | 0.09% | 0.01% | 0.00% | 0.00% | 0.00% | 0.00% |
| **Firmicutes;Clostridia;Clostridiales;Lachnospiraceae;Oribacterium** | 0.91% | 0.70% | 0.79% | 0.02% | 0.00% | 0.00% | 0.00% | 0.00% |
| **Firmicutes;Clostridia;Clostridiales;Lachnospiraceae;Roseburia** | 0.09% | 0.51% | 0.30% | 0.87% | 1.26% | 0.98% | 0.83% | 0.82% |
| **Firmicutes;Clostridia;Clostridiales;Lachnospiraceae;unclassified** | 1.47% | 3.01% | 2.72% | 10.02% | 10.90% | 10.35% | 11.37% | 6.96% |
| **Firmicutes;Clostridia;Clostridiales;Peptostreptococcaceae;Clostridium_XI** | 0.05% | 0.07% | 0.08% | 0.35% | 0.39% | 0.34% | 0.29% | 1.23% |
| **Firmicutes;Clostridia;Clostridiales;Peptostreptococcaceae;Filifactor** | 0.07% | 0.08% | 0.02% | 0.00% | 0.00% | 0.00% | 0.00% | 0.00% |
| **Firmicutes;Clostridia;Clostridiales;Peptostreptococcaceae;Peptostreptococcus** | 0.14% | 0.17% | 0.19% | 0.04% | 0.00% | 0.01% | 0.01% | 0.00% |
| **Firmicutes;Clostridia;Clostridiales;Peptostreptococcaceae;unclassified** | 0.00% | 0.01% | 0.00% | 0.02% | 0.00% | 0.01% | 0.00% | 0.09% |
| **Firmicutes;Clostridia;Clostridiales;Ruminococcaceae;Butyricicoccus** | 0.00% | 0.00% | 0.00% | 0.06% | 0.07% | 0.14% | 0.07% | 0.06% |
| **Firmicutes;Clostridia;Clostridiales;Ruminococcaceae;Clostridium_IV** | 0.00% | 0.00% | 0.00% | 0.01% | 0.00% | 0.00% | 0.01% | 0.03% |
| **Firmicutes;Clostridia;Clostridiales;Ruminococcaceae;Faecalibacterium** | 0.34% | 1.18% | 1.26% | 6.44% | 7.12% | 6.92% | 6.29% | 5.78% |
| **Firmicutes;Clostridia;Clostridiales;Ruminococcaceae;Flavonifractor** | 0.00% | 0.00% | 0.00% | 0.06% | 0.06% | 0.07% | 0.04% | 0.18% |
| **Firmicutes;Clostridia;Clostridiales;Ruminococcaceae;Oscillibacter** | 0.01% | 0.10% | 0.03% | 0.21% | 0.12% | 0.17% | 0.16% | 0.50% |
| **Firmicutes;Clostridia;Clostridiales;Ruminococcaceae;Pseudoflavonifractor** | 0.00% | 0.00% | 0.00% | 0.01% | 0.01% | 0.00% | 0.01% | 0.07% |
| **Firmicutes;Clostridia;Clostridiales;Ruminococcaceae;Ruminococcus** | 0.01% | 0.00% | 0.00% | 0.01% | 0.01% | 0.00% | 0.03% | 0.07% |
| **Firmicutes;Clostridia;Clostridiales;Ruminococcaceae;Subdoligranulum** | 0.00% | 0.00% | 0.01% | 0.01% | 0.01% | 0.02% | 0.02% | 0.03% |
| **Firmicutes;Clostridia;Clostridiales;Ruminococcaceae;unclassified** | 0.03% | 0.22% | 0.09% | 0.42% | 0.60% | 0.39% | 0.61% | 3.11% |
| **Firmicutes;Clostridia;Clostridiales;unclassified;unclassified** | 0.35% | 0.26% | 0.30% | 0.52% | 0.49% | 0.48% | 0.54% | 1.07% |
| **Firmicutes;Clostridia;unclassified;unclassified;unclassified** | 0.00% | 0.01% | 0.03% | 0.01% | 0.07% | 0.02% | 0.00% | 0.03% |
| **Firmicutes;Erysipelotrichia;Erysipelotrichales;Erysipelotrichaceae;Bulleidia** | 0.00% | 0.00% | 0.00% | 0.01% | 0.00% | 0.00% | 0.00% | 0.00% |
| **Firmicutes;Erysipelotrichia;Erysipelotrichales;Erysipelotrichaceae;Catenibacterium** | 0.00% | 0.03% | 0.00% | 0.01% | 0.02% | 0.03% | 0.02% | 0.05% |
| **Firmicutes;Erysipelotrichia;Erysipelotrichales;Erysipelotrichaceae;Clostridium_XVIII** | 0.03% | 0.04% | 0.01% | 0.55% | 0.62% | 0.69% | 0.40% | 0.07% |
| **Firmicutes;Erysipelotrichia;Erysipelotrichales;Erysipelotrichaceae;Coprobacillus** | 0.00% | 0.00% | 0.00% | 0.00% | 0.00% | 0.00% | 0.00% | 0.03% |
| **Firmicutes;Erysipelotrichia;Erysipelotrichales;Erysipelotrichaceae;Erysipelothrix** | 0.03% | 0.00% | 0.00% | 0.00% | 0.00% | 0.00% | 0.00% | 0.00% |
| **Firmicutes;Erysipelotrichia;Erysipelotrichales;Erysipelotrichaceae;Erysipelotrichaceae_incertae_sedis** | 0.01% | 0.30% | 0.02% | 1.57% | 1.41% | 1.31% | 1.16% | 1.04% |
| **Firmicutes;Erysipelotrichia;Erysipelotrichales;Erysipelotrichaceae;Holdemania** | 0.00% | 0.00% | 0.03% | 0.10% | 0.03% | 0.03% | 0.03% | 0.10% |
| **Firmicutes;Erysipelotrichia;Erysipelotrichales;Erysipelotrichaceae;Solobacterium** | 0.61% | 0.46% | 0.48% | 0.07% | 0.01% | 0.01% | 0.01% | 0.00% |
| **Firmicutes;Erysipelotrichia;Erysipelotrichales;Erysipelotrichaceae;Turicibacter** | 0.00% | 0.00% | 0.03% | 0.07% | 0.01% | 0.01% | 0.08% | 0.03% |
| **Firmicutes;Erysipelotrichia;Erysipelotrichales;Erysipelotrichaceae;unclassified** | 0.08% | 0.20% | 0.40% | 0.69% | 0.73% | 0.73% | 0.58% | 0.82% |
| **Firmicutes;Negativicutes;Selenomonadales;Acidaminococcaceae;Acidaminococcus** | 0.00% | 0.00% | 0.00% | 0.00% | 0.00% | 0.00% | 0.01% | 0.00% |
| **Firmicutes;Negativicutes;Selenomonadales;Acidaminococcaceae;Phascolarctobacterium** | 0.01% | 0.10% | 0.04% | 0.28% | 0.40% | 0.33% | 0.41% | 0.77% |
| **Firmicutes;Negativicutes;Selenomonadales;Acidaminococcaceae;unclassified** | 0.05% | 0.10% | 0.10% | 0.01% | 0.00% | 0.00% | 0.00% | 0.00% |
| **Firmicutes;Negativicutes;Selenomonadales;Veillonellaceae;Allisonella** | 0.00% | 0.01% | 0.00% | 0.00% | 0.01% | 0.00% | 0.00% | 0.01% |
| **Firmicutes;Negativicutes;Selenomonadales;Veillonellaceae;Dialister** | 0.10% | 0.13% | 0.10% | 0.13% | 0.24% | 0.20% | 0.20% | 1.48% |
| **Firmicutes;Negativicutes;Selenomonadales;Veillonellaceae;Megamonas** | 0.01% | 0.00% | 0.00% | 0.00% | 0.00% | 0.00% | 0.00% | 0.00% |
| **Firmicutes;Negativicutes;Selenomonadales;Veillonellaceae;Megasphaera** | 0.50% | 0.40% | 0.42% | 0.04% | 0.00% | 0.00% | 0.00% | 0.01% |
| **Firmicutes;Negativicutes;Selenomonadales;Veillonellaceae;Mitsuokella** | 0.00% | 0.00% | 0.00% | 0.00% | 0.01% | 0.00% | 0.00% | 0.00% |
| **Firmicutes;Negativicutes;Selenomonadales;Veillonellaceae;Selenomonas** | 0.16% | 0.08% | 0.15% | 0.00% | 0.00% | 0.00% | 0.00% | 0.00% |
| **Firmicutes;Negativicutes;Selenomonadales;Veillonellaceae;Veillonella** | 5.52% | 5.15% | 3.97% | 0.26% | 0.22% | 0.14% | 0.18% | 0.01% |
| **Firmicutes;Negativicutes;Selenomonadales;Veillonellaceae;Veillonellaceae_genus_incertae_sedis** | 0.01% | 0.00% | 0.03% | 0.00% | 0.00% | 0.00% | 0.00% | 0.00% |
| **Firmicutes;Negativicutes;Selenomonadales;Veillonellaceae;Zymophilus** | 0.01% | 0.01% | 0.02% | 0.00% | 0.00% | 0.00% | 0.00% | 0.00% |
| **Firmicutes;Negativicutes;Selenomonadales;Veillonellaceae;unclassified** | 0.14% | 0.24% | 0.03% | 0.20% | 0.15% | 0.11% | 0.10% | 0.10% |
| **Firmicutes;unclassified;unclassified;unclassified;unclassified** | 0.11% | 0.05% | 0.03% | 0.25% | 0.37% | 0.27% | 0.25% | 0.15% |
| **Fusobacteria;Fusobacteria;Fusobacteriales;Fusobacteriaceae;Fusobacterium** | 2.58% | 2.31% | 2.31% | 0.16% | 0.05% | 0.03% | 0.09% | 0.01% |
| **Fusobacteria;Fusobacteria;Fusobacteriales;Leptotrichiaceae;Leptotrichia** | 1.45% | 1.35% | 0.68% | 0.00% | 0.00% | 0.00% | 0.01% | 0.00% |
| **Fusobacteria;Fusobacteria;Fusobacteriales;Leptotrichiaceae;Sneathia** | 0.04% | 0.00% | 0.00% | 0.00% | 0.00% | 0.00% | 0.00% | 0.00% |
| **Fusobacteria;Fusobacteria;Fusobacteriales;Leptotrichiaceae;Streptobacillus** | 0.05% | 0.03% | 0.00% | 0.00% | 0.00% | 0.00% | 0.00% | 0.00% |
| **Fusobacteria;Fusobacteria;Fusobacteriales;unclassified;unclassified** | 0.24% | 0.29% | 0.26% | 0.00% | 0.00% | 0.00% | 0.00% | 0.00% |
| **Proteobacteria;Alphaproteobacteria;Caulobacterales;Caulobacteraceae;Brevundimonas** | 0.07% | 0.01% | 0.05% | 0.00% | 0.00% | 0.00% | 0.00% | 0.00% |
| **Proteobacteria;Alphaproteobacteria;Caulobacterales;Caulobacteraceae;Caulobacter** | 0.03% | 0.00% | 0.03% | 0.00% | 0.00% | 0.00% | 0.00% | 0.00% |
| **Proteobacteria;Alphaproteobacteria;Rhizobiales;Aurantimonadaceae;Aurantimonas** | 0.01% | 0.00% | 0.00% | 0.00% | 0.00% | 0.00% | 0.00% | 0.00% |
| **Proteobacteria;Alphaproteobacteria;Rhizobiales;Beijerinckiaceae;Chelatococcus** | 0.02% | 0.00% | 0.00% | 0.00% | 0.00% | 0.00% | 0.00% | 0.00% |
| **Proteobacteria;Alphaproteobacteria;Rhizobiales;Bradyrhizobiaceae;Bradyrhizobium** | 0.10% | 0.00% | 0.02% | 0.00% | 0.00% | 0.00% | 0.00% | 0.00% |
| **Proteobacteria;Alphaproteobacteria;Rhizobiales;Bradyrhizobiaceae;unclassified** | 0.00% | 0.01% | 0.00% | 0.00% | 0.00% | 0.00% | 0.00% | 0.00% |
| **Proteobacteria;Alphaproteobacteria;Rhizobiales;Brucellaceae;Daeguia** | 0.00% | 0.01% | 0.00% | 0.00% | 0.00% | 0.00% | 0.00% | 0.00% |
| **Proteobacteria;Alphaproteobacteria;Rhizobiales;Brucellaceae;Ochrobactrum** | 0.00% | 0.02% | 0.05% | 0.00% | 0.00% | 0.01% | 0.00% | 0.00% |
| **Proteobacteria;Alphaproteobacteria;Rhizobiales;Hyphomicrobiaceae;Devosia** | 0.00% | 0.00% | 0.01% | 0.00% | 0.00% | 0.01% | 0.00% | 0.00% |
| **Proteobacteria;Alphaproteobacteria;Rhizobiales;Methylobacteriaceae;Methylobacterium** | 0.02% | 0.11% | 0.05% | 0.00% | 0.00% | 0.00% | 0.00% | 0.00% |
| **Proteobacteria;Alphaproteobacteria;Rhizobiales;Rhizobiaceae;Rhizobium** | 0.03% | 0.00% | 0.02% | 0.00% | 0.00% | 0.00% | 0.00% | 0.00% |
| **Proteobacteria;Alphaproteobacteria;Rhizobiales;unclassified;unclassified** | 0.03% | 0.03% | 0.03% | 0.00% | 0.00% | 0.00% | 0.00% | 0.00% |
| **Proteobacteria;Alphaproteobacteria;Rhodobacterales;Rhodobacteraceae;Haematobacter** | 0.01% | 0.00% | 0.00% | 0.00% | 0.00% | 0.00% | 0.00% | 0.00% |
| **Proteobacteria;Alphaproteobacteria;Rhodobacterales;Rhodobacteraceae;Paracoccus** | 0.03% | 0.00% | 0.03% | 0.00% | 0.00% | 0.00% | 0.00% | 0.00% |
| **Proteobacteria;Alphaproteobacteria;Rhodobacterales;Rhodobacteraceae;unclassified** | 0.01% | 0.01% | 0.00% | 0.00% | 0.00% | 0.00% | 0.00% | 0.00% |
| **Proteobacteria;Alphaproteobacteria;Sphingomonadales;Sphingomonadaceae;Novosphingobium** | 0.00% | 0.04% | 0.29% | 0.00% | 0.00% | 0.00% | 0.01% | 0.00% |
| **Proteobacteria;Alphaproteobacteria;Sphingomonadales;Sphingomonadaceae;Sphingobium** | 0.03% | 0.00% | 0.00% | 0.00% | 0.00% | 0.01% | 0.00% | 0.00% |
| **Proteobacteria;Alphaproteobacteria;Sphingomonadales;Sphingomonadaceae;Sphingomonas** | 0.04% | 0.01% | 0.28% | 0.00% | 0.00% | 0.00% | 0.00% | 0.00% |
| **Proteobacteria;Alphaproteobacteria;Sphingomonadales;Sphingomonadaceae;unclassified** | 0.06% | 0.01% | 0.02% | 0.00% | 0.00% | 0.00% | 0.00% | 0.00% |
| **Proteobacteria;Alphaproteobacteria;Sphingomonadales;unclassified;unclassified** | 0.01% | 0.00% | 0.00% | 0.00% | 0.00% | 0.00% | 0.00% | 0.00% |
| **Proteobacteria;Alphaproteobacteria;unclassified;unclassified;unclassified** | 0.00% | 0.00% | 0.07% | 0.00% | 0.00% | 0.00% | 0.01% | 0.00% |
| **Proteobacteria;Betaproteobacteria;Burkholderiales;Alcaligenaceae;Achromobacter** | 0.00% | 0.01% | 0.00% | 0.00% | 0.00% | 0.00% | 0.00% | 0.00% |
| **Proteobacteria;Betaproteobacteria;Burkholderiales;Burkholderiaceae;Cupriavidus** | 0.03% | 0.01% | 0.00% | 0.00% | 0.00% | 0.00% | 0.00% | 0.00% |
| **Proteobacteria;Betaproteobacteria;Burkholderiales;Burkholderiaceae;Ralstonia** | 0.87% | 0.82% | 1.03% | 0.00% | 0.00% | 0.00% | 0.01% | 0.00% |
| **Proteobacteria;Betaproteobacteria;Burkholderiales;Burkholderiales_incertae_sedis;Aquabacterium** | 0.00% | 0.02% | 0.01% | 0.00% | 0.00% | 0.00% | 0.01% | 0.00% |
| **Proteobacteria;Betaproteobacteria;Burkholderiales;Burkholderiales_incertae_sedis;Ideonella** | 0.01% | 0.00% | 0.00% | 0.00% | 0.00% | 0.00% | 0.00% | 0.00% |
| **Proteobacteria;Betaproteobacteria;Burkholderiales;Burkholderiales_incertae_sedis;Tepidimonas** | 0.15% | 0.01% | 0.01% | 0.00% | 0.00% | 0.00% | 0.00% | 0.00% |
| **Proteobacteria;Betaproteobacteria;Burkholderiales;Burkholderiales_incertae_sedis;unclassified** | 0.01% | 0.00% | 0.01% | 0.00% | 0.00% | 0.00% | 0.00% | 0.00% |
| **Proteobacteria;Betaproteobacteria;Burkholderiales;Comamonadaceae;Acidovorax** | 0.03% | 0.04% | 0.04% | 0.00% | 0.00% | 0.00% | 0.00% | 0.00% |
| **Proteobacteria;Betaproteobacteria;Burkholderiales;Comamonadaceae;Comamonas** | 0.07% | 0.08% | 0.01% | 0.00% | 0.00% | 0.00% | 0.00% | 0.00% |
| **Proteobacteria;Betaproteobacteria;Burkholderiales;Comamonadaceae;Delftia** | 0.05% | 0.00% | 0.00% | 0.00% | 0.00% | 0.00% | 0.00% | 0.00% |
| **Proteobacteria;Betaproteobacteria;Burkholderiales;Comamonadaceae;Diaphorobacter** | 0.03% | 0.00% | 0.08% | 0.00% | 0.00% | 0.00% | 0.00% | 0.00% |
| **Proteobacteria;Betaproteobacteria;Burkholderiales;Comamonadaceae;Pelomonas** | 0.06% | 0.12% | 0.16% | 0.00% | 0.01% | 0.00% | 0.00% | 0.00% |
| **Proteobacteria;Betaproteobacteria;Burkholderiales;Comamonadaceae;Schlegelella** | 0.06% | 0.12% | 0.10% | 0.00% | 0.00% | 0.00% | 0.00% | 0.00% |
| **Proteobacteria;Betaproteobacteria;Burkholderiales;Comamonadaceae;Variovorax** | 0.20% | 0.12% | 0.16% | 0.00% | 0.00% | 0.00% | 0.00% | 0.00% |
| **Proteobacteria;Betaproteobacteria;Burkholderiales;Comamonadaceae;unclassified** | 0.00% | 0.00% | 0.01% | 0.00% | 0.00% | 0.00% | 0.00% | 0.00% |
| **Proteobacteria;Betaproteobacteria;Burkholderiales;Oxalobacteraceae;Herbaspirillum** | 0.00% | 0.01% | 0.00% | 0.00% | 0.00% | 0.00% | 0.00% | 0.00% |
| **Proteobacteria;Betaproteobacteria;Burkholderiales;Oxalobacteraceae;Janthinobacterium** | 0.16% | 0.16% | 0.00% | 0.00% | 0.01% | 0.01% | 0.00% | 0.00% |
| **Proteobacteria;Betaproteobacteria;Burkholderiales;Oxalobacteraceae;Massilia** | 0.00% | 0.01% | 0.00% | 0.00% | 0.00% | 0.00% | 0.00% | 0.00% |
| **Proteobacteria;Betaproteobacteria;Burkholderiales;Oxalobacteraceae;Undibacterium** | 0.04% | 0.04% | 0.10% | 0.00% | 0.00% | 0.00% | 0.00% | 0.00% |
| **Proteobacteria;Betaproteobacteria;Burkholderiales;Sutterellaceae;Parasutterella** | 0.00% | 0.00% | 0.02% | 0.12% | 0.18% | 0.10% | 0.18% | 0.14% |
| **Proteobacteria;Betaproteobacteria;Burkholderiales;Sutterellaceae;Sutterella** | 0.05% | 0.08% | 0.06% | 0.98% | 0.70% | 0.45% | 0.86% | 0.28% |
| **Proteobacteria;Betaproteobacteria;Burkholderiales;Sutterellaceae;unclassified** | 0.00% | 0.00% | 0.00% | 0.06% | 0.13% | 0.16% | 0.10% | 0.12% |
| **Proteobacteria;Betaproteobacteria;Burkholderiales;unclassified;unclassified** | 0.03% | 0.01% | 0.01% | 0.04% | 0.01% | 0.05% | 0.07% | 0.03% |
| **Proteobacteria;Betaproteobacteria;Hydrogenophilales;Hydrogenophilaceae;Hydrogenophilus** | 0.01% | 0.00% | 0.00% | 0.00% | 0.00% | 0.00% | 0.00% | 0.00% |
| **Proteobacteria;Betaproteobacteria;Hydrogenophilales;Hydrogenophilaceae;Petrobacter** | 0.01% | 0.00% | 0.00% | 0.00% | 0.00% | 0.00% | 0.00% | 0.00% |
| **Proteobacteria;Betaproteobacteria;Hydrogenophilales;Hydrogenophilaceae;Thiobacillus** | 0.00% | 0.00% | 0.01% | 0.00% | 0.00% | 0.00% | 0.00% | 0.00% |
| **Proteobacteria;Betaproteobacteria;Neisseriales;Neisseriaceae;Aquitalea** | 0.01% | 0.00% | 0.00% | 0.00% | 0.00% | 0.00% | 0.00% | 0.00% |
| **Proteobacteria;Betaproteobacteria;Neisseriales;Neisseriaceae;Eikenella** | 0.00% | 0.01% | 0.01% | 0.00% | 0.00% | 0.00% | 0.00% | 0.00% |
| **Proteobacteria;Betaproteobacteria;Neisseriales;Neisseriaceae;Kingella** | 0.12% | 0.00% | 0.02% | 0.00% | 0.00% | 0.00% | 0.00% | 0.00% |
| **Proteobacteria;Betaproteobacteria;Neisseriales;Neisseriaceae;Neisseria** | 0.50% | 0.46% | 0.37% | 0.00% | 0.00% | 0.00% | 0.00% | 0.00% |
| **Proteobacteria;Betaproteobacteria;Neisseriales;Neisseriaceae;Simonsiella** | 0.00% | 0.00% | 0.01% | 0.00% | 0.00% | 0.00% | 0.00% | 0.00% |
| **Proteobacteria;Betaproteobacteria;Neisseriales;Neisseriaceae;unclassified** | 0.07% | 0.07% | 0.17% | 0.00% | 0.00% | 0.00% | 0.00% | 0.00% |
| **Proteobacteria;Betaproteobacteria;Rhodocyclales;Rhodocyclaceae;Azospira** | 0.00% | 0.02% | 0.00% | 0.00% | 0.00% | 0.00% | 0.00% | 0.00% |
| **Proteobacteria;Betaproteobacteria;Rhodocyclales;Rhodocyclaceae;Quatrionicoccus** | 0.03% | 0.00% | 0.00% | 0.00% | 0.00% | 0.00% | 0.00% | 0.00% |
| **Proteobacteria;Betaproteobacteria;unclassified;unclassified;unclassified** | 0.00% | 0.03% | 0.00% | 0.11% | 0.16% | 0.12% | 0.16% | 0.12% |
| **Proteobacteria;Deltaproteobacteria;Desulfovibrionales;Desulfovibrionaceae;Bilophila** | 0.00% | 0.00% | 0.00% | 0.00% | 0.00% | 0.01% | 0.01% | 0.01% |
| **Proteobacteria;Deltaproteobacteria;Desulfovibrionales;Desulfovibrionaceae;Desulfovibrio** | 0.00% | 0.04% | 0.00% | 0.01% | 0.01% | 0.01% | 0.00% | 0.01% |
| **Proteobacteria;Deltaproteobacteria;Desulfovibrionales;Desulfovibrionaceae;unclassified** | 0.00% | 0.01% | 0.00% | 0.01% | 0.02% | 0.01% | 0.01% | 0.01% |
| **Proteobacteria;Deltaproteobacteria;unclassified;unclassified;unclassified** | 0.00% | 0.00% | 0.02% | 0.02% | 0.01% | 0.01% | 0.05% | 0.01% |
| **Proteobacteria;Epsilonproteobacteria;Campylobacterales;Campylobacteraceae;Arcobacter** | 0.00% | 0.00% | 0.01% | 0.00% | 0.00% | 0.00% | 0.00% | 0.00% |
| **Proteobacteria;Epsilonproteobacteria;Campylobacterales;Campylobacteraceae;Campylobacter** | 0.31% | 0.29% | 0.21% | 0.01% | 0.00% | 0.01% | 0.00% | 0.00% |
| **Proteobacteria;Epsilonproteobacteria;Campylobacterales;Campylobacteraceae;Sulfurospirillum** | 0.03% | 0.10% | 0.03% | 0.00% | 0.00% | 0.00% | 0.01% | 0.00% |
| **Proteobacteria;Epsilonproteobacteria;Campylobacterales;Helicobacteraceae;Helicobacter** | 0.48% | 0.07% | 0.16% | 0.00% | 0.02% | 0.02% | 0.01% | 0.03% |
| **Proteobacteria;Gammaproteobacteria;Aeromonadales;Aeromonadaceae;unclassified** | 0.01% | 0.00% | 0.00% | 0.00% | 0.00% | 0.00% | 0.00% | 0.00% |
| **Proteobacteria;Gammaproteobacteria;Aeromonadales;Succinivibrionaceae;Succinivibrio** | 0.00% | 0.07% | 0.00% | 0.04% | 0.03% | 0.00% | 0.08% | 0.28% |
| **Proteobacteria;Gammaproteobacteria;Alteromonadales;Alteromonadaceae;Alishewanella** | 0.00% | 0.00% | 0.01% | 0.00% | 0.00% | 0.00% | 0.00% | 0.00% |
| **Proteobacteria;Gammaproteobacteria;Cardiobacteriales;Cardiobacteriaceae;Cardiobacterium** | 0.00% | 0.01% | 0.01% | 0.00% | 0.00% | 0.00% | 0.00% | 0.00% |
| **Proteobacteria;Gammaproteobacteria;Enterobacteriales;Enterobacteriaceae;Buttiauxella** | 0.03% | 0.01% | 0.03% | 0.00% | 0.00% | 0.00% | 0.00% | 0.00% |
| **Proteobacteria;Gammaproteobacteria;Enterobacteriales;Enterobacteriaceae;Citrobacter** | 0.00% | 0.00% | 0.00% | 0.01% | 0.02% | 0.02% | 0.04% | 0.10% |
| **Proteobacteria;Gammaproteobacteria;Enterobacteriales;Enterobacteriaceae;Enterobacter** | 0.00% | 0.00% | 0.00% | 0.00% | 0.00% | 0.00% | 0.00% | 0.01% |
| **Proteobacteria;Gammaproteobacteria;Enterobacteriales;Enterobacteriaceae;Escherichia_Shigella** | 1.11% | 1.38% | 1.48% | 0.93% | 0.72% | 0.66% | 0.84% | 1.28% |
| **Proteobacteria;Gammaproteobacteria;Enterobacteriales;Enterobacteriaceae;Klebsiella** | 0.00% | 0.00% | 0.00% | 0.00% | 0.00% | 0.00% | 0.01% | 0.00% |
| **Proteobacteria;Gammaproteobacteria;Enterobacteriales;Enterobacteriaceae;Kluyvera** | 0.03% | 0.00% | 0.03% | 0.00% | 0.00% | 0.00% | 0.00% | 0.00% |
| **Proteobacteria;Gammaproteobacteria;Enterobacteriales;Enterobacteriaceae;Morganella** | 0.01% | 0.00% | 0.00% | 0.00% | 0.00% | 0.00% | 0.00% | 0.00% |
| **Proteobacteria;Gammaproteobacteria;Enterobacteriales;Enterobacteriaceae;Serratia** | 0.03% | 0.12% | 0.08% | 0.01% | 0.00% | 0.00% | 0.01% | 0.00% |
| **Proteobacteria;Gammaproteobacteria;Enterobacteriales;Enterobacteriaceae;Yersinia** | 0.01% | 0.03% | 0.02% | 0.00% | 0.00% | 0.00% | 0.01% | 0.00% |
| **Proteobacteria;Gammaproteobacteria;Enterobacteriales;Enterobacteriaceae;unclassified** | 2.02% | 2.72% | 3.11% | 0.10% | 0.11% | 0.10% | 0.12% | 0.23% |
| **Proteobacteria;Gammaproteobacteria;Oceanospirillales;Halomonadaceae;Chromohalobacter** | 0.00% | 0.00% | 0.01% | 0.00% | 0.00% | 0.00% | 0.00% | 0.00% |
| **Proteobacteria;Gammaproteobacteria;Oceanospirillales;Halomonadaceae;Halomonas** | 0.03% | 0.07% | 0.03% | 0.00% | 0.00% | 0.00% | 0.00% | 0.00% |
| **Proteobacteria;Gammaproteobacteria;Pasteurellales;Pasteurellaceae;Actinobacillus** | 0.45% | 0.12% | 0.12% | 0.06% | 0.00% | 0.00% | 0.00% | 0.00% |
| **Proteobacteria;Gammaproteobacteria;Pasteurellales;Pasteurellaceae;Aggregatibacter** | 0.03% | 0.00% | 0.01% | 0.00% | 0.00% | 0.00% | 0.00% | 0.00% |
| **Proteobacteria;Gammaproteobacteria;Pasteurellales;Pasteurellaceae;Haemophilus** | 0.48% | 0.33% | 0.31% | 0.10% | 0.07% | 0.07% | 0.08% | 0.00% |
| **Proteobacteria;Gammaproteobacteria;Pasteurellales;Pasteurellaceae;unclassified** | 0.50% | 0.46% | 0.67% | 0.11% | 0.06% | 0.06% | 0.07% | 0.00% |
| **Proteobacteria;Gammaproteobacteria;Pseudomonadales;Moraxellaceae;Acinetobacter** | 0.04% | 0.08% | 0.07% | 0.00% | 0.00% | 0.00% | 0.00% | 0.05% |
| **Proteobacteria;Gammaproteobacteria;Pseudomonadales;Moraxellaceae;Alkanindiges** | 0.00% | 0.01% | 0.00% | 0.00% | 0.00% | 0.00% | 0.00% | 0.00% |
| **Proteobacteria;Gammaproteobacteria;Pseudomonadales;Moraxellaceae;Enhydrobacter** | 0.05% | 0.00% | 0.02% | 0.00% | 0.00% | 0.00% | 0.00% | 0.00% |
| **Proteobacteria;Gammaproteobacteria;Pseudomonadales;Moraxellaceae;Moraxella** | 0.00% | 0.01% | 0.00% | 0.00% | 0.00% | 0.00% | 0.00% | 0.00% |
| **Proteobacteria;Gammaproteobacteria;Pseudomonadales;Moraxellaceae;Psychrobacter** | 0.00% | 0.00% | 0.05% | 0.00% | 0.00% | 0.00% | 0.00% | 0.00% |
| **Proteobacteria;Gammaproteobacteria;Pseudomonadales;Pseudomonadaceae;Cellvibrio** | 0.01% | 0.00% | 0.00% | 0.00% | 0.00% | 0.00% | 0.00% | 0.00% |
| **Proteobacteria;Gammaproteobacteria;Pseudomonadales;Pseudomonadaceae;Pseudomonas** | 8.40% | 8.84% | 9.87% | 0.13% | 0.07% | 0.18% | 0.20% | 0.00% |
| **Proteobacteria;Gammaproteobacteria;Pseudomonadales;Pseudomonadaceae;Rhizobacter** | 0.00% | 0.00% | 0.07% | 0.00% | 0.00% | 0.00% | 0.00% | 0.00% |
| **Proteobacteria;Gammaproteobacteria;Pseudomonadales;Pseudomonadaceae;unclassified** | 0.01% | 0.01% | 0.01% | 0.00% | 0.00% | 0.00% | 0.00% | 0.00% |
| **Proteobacteria;Gammaproteobacteria;Xanthomonadales;Sinobacteraceae;Nevskia** | 0.01% | 0.00% | 0.00% | 0.00% | 0.00% | 0.00% | 0.00% | 0.00% |
| **Proteobacteria;Gammaproteobacteria;Xanthomonadales;Xanthomonadaceae;Lysobacter** | 0.00% | 0.01% | 0.01% | 0.00% | 0.00% | 0.00% | 0.00% | 0.00% |
| **Proteobacteria;Gammaproteobacteria;Xanthomonadales;Xanthomonadaceae;Stenotrophomonas** | 0.09% | 0.10% | 0.22% | 0.00% | 0.01% | 0.00% | 0.00% | 0.00% |
| **Proteobacteria;Gammaproteobacteria;Xanthomonadales;Xanthomonadaceae;Thermomonas** | 0.01% | 0.00% | 0.00% | 0.00% | 0.00% | 0.00% | 0.00% | 0.00% |
| **Proteobacteria;Gammaproteobacteria;Xanthomonadales;Xanthomonadaceae;unclassified** | 0.01% | 0.01% | 0.00% | 0.00% | 0.00% | 0.00% | 0.00% | 0.00% |
| **Proteobacteria;Gammaproteobacteria;unclassified;unclassified;unclassified** | 0.01% | 0.03% | 0.01% | 0.00% | 0.00% | 0.00% | 0.00% | 0.00% |
| **Proteobacteria;unclassified;unclassified;unclassified;unclassified** | 0.00% | 0.00% | 0.00% | 0.06% | 0.08% | 0.07% | 0.18% | 0.07% |
| **SR1** | 0.07% | 0.09% | 0.10% | 0.00% | 0.00% | 0.00% | 0.00% | 0.00% |
| **Spirochaetes;Spirochaetes;Spirochaetales;Spirochaetaceae;Treponema** | 0.20% | 0.05% | 0.14% | 0.00% | 0.00% | 0.00% | 0.00% | 0.00% |
| **TM7** | 0.57% | 0.54% | 0.49% | 0.02% | 0.00% | 0.01% | 0.01% | 0.00% |
| **Tenericutes;Mollicutes;Mycoplasmatales;Mycoplasmataceae;Mycoplasma** | 0.05% | 0.01% | 0.01% | 0.00% | 0.00% | 0.00% | 0.00% | 0.00% |
| **Verrucomicrobia;Verrucomicrobiae;Verrucomicrobiales;Verrucomicrobiaceae;Akkermansia** | 0.00% | 0.00% | 0.00% | 0.00% | 0.00% | 0.01% | 0.01% | 0.01% |
| **Bacteria;unclassified;unclassified;unclassified;unclassified;unclassified** | 0.67% | 0.83% | 0.73% | 1.66% | 1.51% | 1.67% | 1.82% | 2.83% |

**S6: Comparing upper GI and stools mean abundance of genera**

| Test-Statistic | FDR corr. *P* | upper mean* | stool mean | taxonomy |
| --- | --- | --- | --- | --- |
| -7.183462401 | 0.01490 | 154.26 | 554.77 | Bacteroidetes;Bacteroidia;Bacteroidales;Bacteroidaceae;Bacteroides |
| 5.651249219 | 0.01490 | 203.23 | 3.46 | Firmicutes;Bacilli;Lactobacillales;Streptococcaceae;Streptococcus |
| -4.830345776 | 0.01490 | 9.97 | 64.77 | Firmicutes;Clostridia;Clostridiales;Lachnospiraceae;Lachnospiracea incertae sedis |
| -6.143188217 | 0.01490 | 10.90 | 68.08 | Firmicutes;Clostridia;Clostridiales;Ruminococcaceae;Faecalibacterium |
| 3.958454884 | 0.01490 | 106.44 | 0.00 | Proteobacteria;Gammaproteobacteria;Pseudomonadales;Pseudomonadaceae;Pseudomonas |
| 4.948102884 | 0.01490 | 57.51 | 0.08 | Firmicutes;Negativicutes;Selenomonadales;Veillonellaceae;Veillonella |
| 4.113187014 | 0.01490 | 28.23 | 0.08 | Fusobacteria;Fusobacteria;Fusobacteriales;Fusobacteriaceae;Fusobacterium |
| -4.302171678 | 0.01490 | 8.74 | 33.38 | unclassified;unclassified;unclassified;unclassified;unclassified |
| -4.176815788 | 0.01490 | 2.72 | 21.77 | Bacteroidetes;Bacteroidia;Bacteroidales;Porphyromonadaceae;Parabacteroides |
| 3.99461268 | 0.01490 | 20.67 | 0.00 | Firmicutes;Bacilli;Lactobacillales;Carnobacteriaceae;Granulicatella |
| -5.167064331 | 0.01490 | 1.33 | 36.62 | Firmicutes;Clostridia;Clostridiales;Ruminococcaceae;unclassified |
| 3.439037558 | 0.01490 | 12.03 | 0.00 | Actinobacteria;Actinobacteria;Actinomycetales;Propionibacteriaceae;Propionibacterium |
| -5.48115343 | 0.01490 | 0.85 | 13.31 | Bacteroidetes;Bacteroidia;Bacteroidales;Rikenellaceae;Alistipes |
| -4.037206985 | 0.01490 | 3.59 | 12.62 | Firmicutes;Clostridia;Clostridiales;unclassified;unclassified |
| -4.141343241 | 0.01490 | 0.82 | 14.54 | Firmicutes;Clostridia;Clostridiales;Peptostreptococcaceae;Clostridium XI |
| 3.787443987 | 0.01490 | 9.41 | 0.00 | Firmicutes;Clostridia;Clostridiales;Lachnospiraceae;Oribacterium |
| -4.119199737 | 0.01490 | 0.59 | 7.38 | Bacteroidetes;Bacteroidia;Bacteroidales;Porphyromonadaceae;Barnesiella |
| 4.355840504 | 0.01490 | 6.36 | 0.00 | Firmicutes;Clostridia;Clostridiales;Eubacteriaceae;Eubacterium |
| -4.030836398 | 0.01490 | 1.31 | 17.46 | Firmicutes;Negativicutes;Selenomonadales;Veillonellaceae;Dialister |
| 3.624964669 | 0.01490 | 4.38 | 0.00 | Proteobacteria;Gammaproteobacteria;Pasteurellales;Pasteurellaceae;Haemophilus |
| -4.8907214 | 0.01490 | 0.59 | 5.85 | Firmicutes;Clostridia;Clostridiales;Ruminococcaceae;Oscillibacter |
| -5.875120889 | 0.01490 | 0.00 | 0.69 | Firmicutes;Clostridia;Clostridiales;Ruminococcaceae;Butyricicoccus |
| -2.514474228 | 0.01490 | 0.00 | 2.15 | Firmicutes;Clostridia;Clostridiales;Ruminococcaceae;Flavonifractor |
| -3.562145536 | 0.02636 | 28.28 | 82.00 | Firmicutes;Clostridia;Clostridiales;Lachnospiraceae;unclassified |
| -4.118569754 | 0.02636 | 0.31 | 2.00 | Bacteroidetes;Bacteroidia;Bacteroidales;Porphyromonadaceae;Odoribacter |
| -3.241018618 | 0.02636 | 0.05 | 1.08 | Firmicutes;Clostridia;Clostridiales;Peptostreptococcaceae;unclassified |
| 3.354133462 | 0.03316 | 30.82 | 2.69 | Proteobacteria;Gammaproteobacteria;Enterobacteriales;Enterobacteriaceae;unclassified |
| 3.445449499 | 0.03316 | 14.82 | 0.08 | Actinobacteria;Actinobacteria;Actinomycetales;Actinomycetaceae;Actinomyces |
| 3.375292365 | 0.03316 | 6.10 | 0.00 | Firmicutes;Erysipelotrichia;Erysipelotrichales;Erysipelotrichaceae;Solobacterium |
| 3.273238834 | 0.03316 | 5.18 | 0.08 | Firmicutes;Negativicutes;Selenomonadales;Veillonellaceae;Megasphaera |
| -3.508232077 | 0.03316 | 0.03 | 0.85 | Firmicutes;Clostridia;Clostridiales;Ruminococcaceae;Ruminococcus |
| 3.838655167 | 0.03916 | 6.31 | 0.00 | TM7;TM7 class;TM7 order;TM7 family;TM7 genus incertae sedis |
| 3.525059741 | 0.03916 | 6.38 | 0.00 | Proteobacteria;Gammaproteobacteria;Pasteurellales;Pasteurellaceae;unclassified |
| -3.407845789 | 0.03916 | 0.08 | 1.69 | Proteobacteria;Betaproteobacteria;Burkholderiales;Sutterellaceae;Parasutterella |
| -3.066883499 | 0.03916 | 0.10 | 1.15 | Firmicutes;Erysipelotrichia;Erysipelotrichales;Erysipelotrichaceae;Holdemania |
| 2.623603767 | 0.04393 | 19.08 | 0.00 | Firmicutes;Bacilli;Bacillales;Bacillales Incertae Sedis XI;Gemella |
| 3.280163193 | 0.04393 | 13.64 | 0.00 | Fusobacteria;Fusobacteria;Fusobacteriales;Leptotrichiaceae;Leptotrichia |
| -3.194837818 | 0.04393 | 0.59 | 9.08 | Firmicutes;Negativicutes;Selenomonadales;Acidaminococcaceae;Phascolarctobacterium |
| 3.511184366 | 0.04393 | 3.18 | 0.00 | Proteobacteria;Epsilonproteobacteria;Campylobacterales;Campylobacteraceae;Campylobacter |

*Upper GI tract (GC; GA, DD)


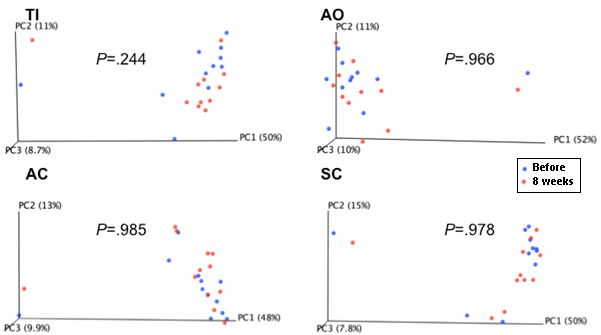


**S7: PCoA-plots generated on a Bray-Curtis distance matrix:** Mucosal samples of the lower GI-tract before (blue dots) and after 8 weeks (red dots) of vitD_3_ supplementation**.** (TI) terminal ileum, (AO) appendiceal orifice, (AC) ascending colon and (SC) sigmoid colon.


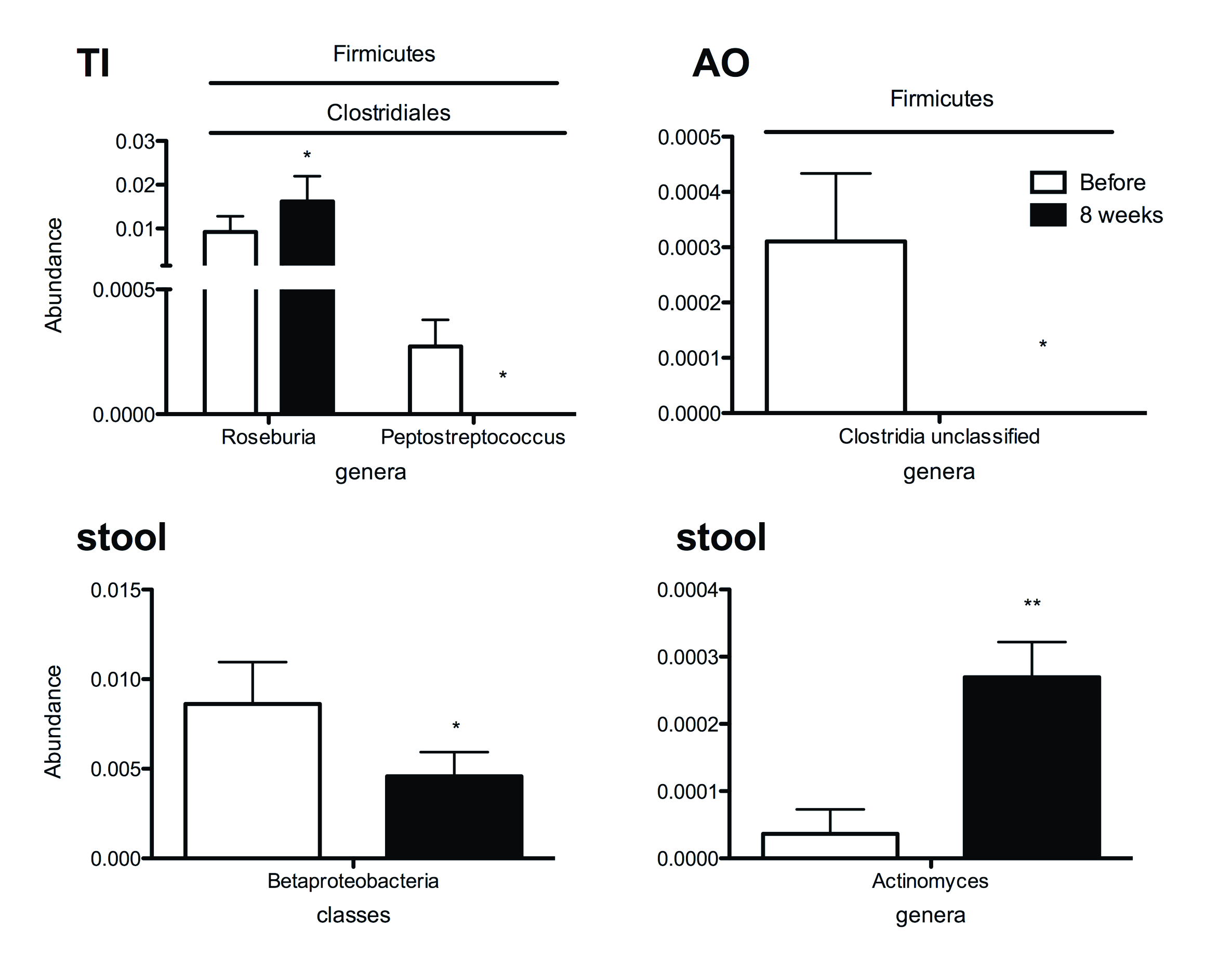


S8: Significantly affected genera and class caused by vitD_3_ treatment in the terminal ileum (TI), appendiceal orifice region (AO), and stool. *P*<.05=*; *P*<.01=**

***P*=.778**

***P*=.327**


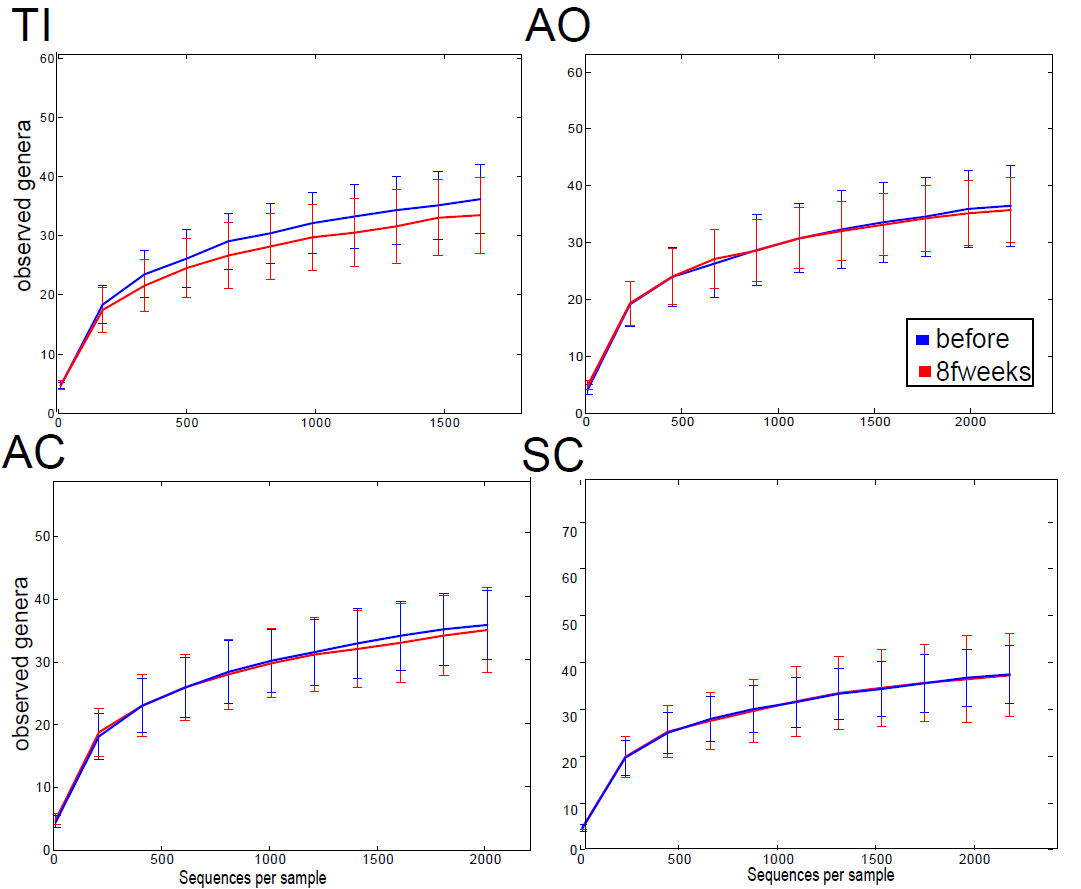


***P*=.951**

***P*=.758**

S9: Alpha rarefaction curves of mucosal samples from the terminal ileum (TI), appendiceal orifice region (AO), ascending colon (AC) and sigmoid colon (SC) before and after 8 weeks of vitD_3_ treatment. No significant changes were observed


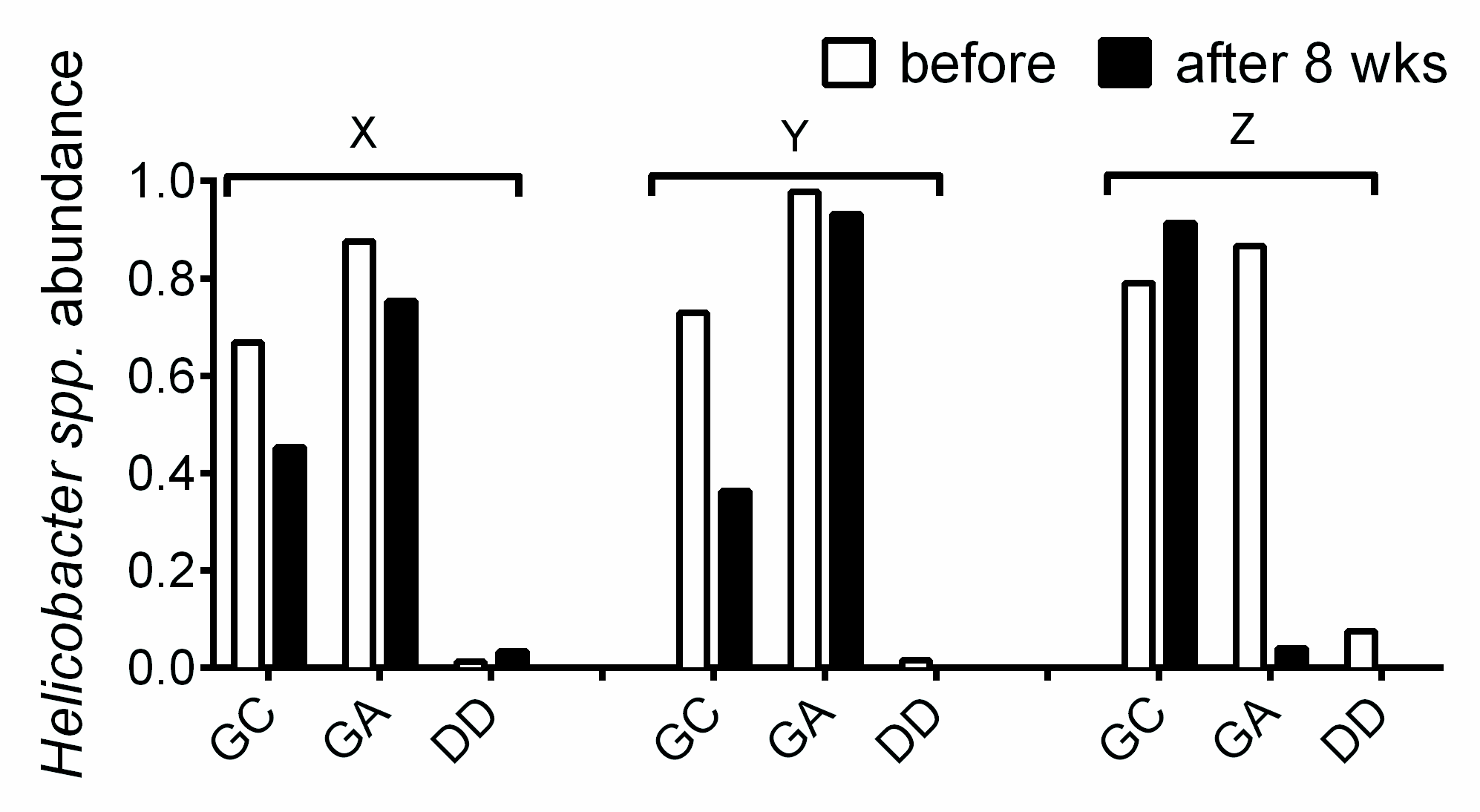


**S10**: **Relative *Helicobacter spp.* abundance in three *H. pylori* positive volunteers.**

Each volunteer is shown separately as the group consists of only three volunteers. In general we see a decline of *Helicobacter spp.* abundance after 8 weeks of vitD_3_ supplementation, except for volunteer X’s duodenum and volunteer Z’s gastric corpus. (GC) gastric corpus, (GA) gastric antrum, (DD) duodenum.
